# Single-cell analysis identifies distinct macrophage phenotypes associated with prodisease and proresolving functions in the endometriotic niche

**DOI:** 10.1073/pnas.2405474121

**Published:** 2024-09-10

**Authors:** Yasmin Henlon, Kavita Panir, Iona McIntyre, Chloe Hogg, Priya Dhami, Antonia O. Cuff, Anna Senior, Niky Moolchandani-Adwani, Elise T. Courtois, Andrew W. Horne, Matthew Rosser, Sascha Ott, Erin Greaves

**Affiliations:** ^a^Division of Biomedical Sciences, Warwick Medical School, University of Warwick, Coventry CV4 7AL, United Kingdom; ^b^Centre for Early Life, University of Warwick, Coventry CV4 7AL, United Kingdom; ^c^Centre for Reproductive Health, Institute of Regeneration and Repair, The University of Edinburgh, Edinburgh EH16 4UU, United Kingdom; ^d^Single Cell Biology Lab, The Jackson Laboratory for Genomic Medicine, Farmington, CT 06032

**Keywords:** endometriosis, macrophage, phenotype, heterogeneity, lesion

## Abstract

Endometriosis negatively impacts the health-related quality of life of 190 million women worldwide. Novel advances in nonhormonal treatments for this debilitating condition are desperately needed. Macrophages play a vital role in the pathophysiology of endometriosis and represent a promising therapeutic target. In the current study, we revealed the full transcriptomic complexity of endometriosis-associated macrophage subpopulations using single-cell analyses in a preclinical mouse model of experimental endometriosis. We have identified two key lesion-resident populations that resemble i) tumor-associated macrophages (characterized by expression of *Folr2*, *Mrc1*, *Gas6,* and *Ccl8+*) that promoted expression of *Col1a1* and *Tgfb1* in human endometrial stromal cells and increased angiogenic meshes in human umbilical vein endothelial cells, and ii) scar-associated macrophages (*Mmp12, Cd9, Spp1, Trem2*+) that exhibited a phenotype associated with fibrosis and matrix remodeling. We also described a population of proresolving large peritoneal macrophages that align with a lipid-associated macrophage phenotype (*Apoe, Saa3, Pid1*) concomitant with altered lipid metabolism and cholesterol efflux. Gain of function experiments using an Apoe mimetic resulted in decreased lesion size and fibrosis, and modification of peritoneal macrophage populations in the preclinical model. Using cross-species analysis of mouse and human single-cell datasets, we determined the concordance of peritoneal and lesion-resident macrophage subpopulations, identifying key similarities and differences in transcriptomic phenotypes. Ultimately, we envisage that these findings will inform the design and use of specific macrophage-targeted therapies and open broad avenues for the treatment of endometriosis.

## Significance

Endometriosis significantly impacts the lives of approximately 190 million women globally. Developing new, nonhormonal treatments is crucial for managing this challenging condition. Macrophages are intimately linked with the pathophysiology of endometriosis and represent promising therapeutic targets. Using single-cell RNA-Seq in a mouse model, we determined the transcriptomic profiles and functions of different macrophages within endometriosis lesions and in the peritoneal cavity. We identified two distinct prodisease lesion-resident phenotypes, resembling tumor-associated macrophages and scar-associated macrophages. Additionally, we characterized a group of protective peritoneal macrophages associated with resolving lesions. We used bioinformatics to compare human and mouse endometriosis lesions and found remarkable concordance between populations. This vital background data will underpin the development of macrophage-directed therapies for endometriosis.

Resident tissue macrophages are integral to the maintenance of healthy tissue function. They exhibit heterogeneity in phenotype and function, and their roles within tissues are dictated by ontogeny, local environment, inflammation status, and time in residence within the microenvironment. These modifying factors mean that each adult tissue contains a unique balance of ontogenetically distinct macrophage populations, and the complement is dynamically modulated throughout life (1). Tissue-resident macrophages in different tissues and at different time-points arise from three different origins: early yolk-sac macrophages, fetal liver monocytes, or bone-marrow-derived monocytes. In some tissues, macrophages of embryonic origin can exist independently of monocyte input and are maintained locally. In other tissues, monocytes continually enter the tissue and replenish macrophage populations (2–4). Inflammatory challenge/injury results in rapid recruitment of monocytes to damaged tissues and a disruption of tissue macrophage homeostasis (1). Peritoneal cavity macrophages play a vital role in immune surveillance of the cavity and visceral organs and are an exemplar of the dynamic mosaic of macrophages in tissues. Two main populations of macrophages exist in the cavity: large (LpM) and small (SpM) peritoneal macrophages. LpM are tissue-resident, abundant, and predominantly embryonically derived in early life. In adulthood, LpM are gradually replenished by monocytes in a sexually dimorphic pattern (5). Although monocyte-derived LpM acquire characteristics of embryo-derived LpM, they exhibit some transcriptional and functional differences (6, 7). Conversely, SpM are less abundant, consist of monocyte-derived macrophages and dendritic cells (DC) and are constantly replenished from infiltrating monocytes (5). These steady-state dynamics are rapidly perturbed by inflammation, that can lead to recruitment of large numbers of inflammatory macrophages and a loss of LpM, the extent of which varies depending on the inflammatory stimuli (7). In parallel to their role in cavity homeostasis, peritoneal macrophages are also central to peritoneal pathologies including endometriosis (8, 9).

Endometriosis is an incurable inflammatory condition characterized by the growth of endometrial-like tissue as “lesions” outside the uterus, usually on the lining of the peritoneal cavity or ovaries. The condition is associated with chronic pelvic pain and infertility and affects approximately 190 million people worldwide (10). Removal of lesions during laparoscopic surgery can relieve symptoms temporarily but recurrence rates are high. Current medical management is via ovarian suppression, which is contraceptive, and symptoms return following cessation of treatment (10). There is an urgent unmet need for new therapeutic targets that may be developed into novel noninvasive, nonhormonal treatments. Disease-modified macrophages represent a promising focus for the development of immune therapies for endometriosis as they become adapted such that they support lesion survival by promoting cell proliferation and vascularization (11). Using our unique syngeneic mouse model of experimental endometriosis (12), we have also demonstrated that macrophages encourage innervation of lesions, sensitization of nerves, and generation of pain (13, 14).

Recently, we identified that macrophages in lesions have different origins and associated functions; they are derived from the eutopic (donor) endometrium, from (host-derived) LpM that traffic into lesions, and monocytes that infiltrate lesions and differentiate into macrophages (15). Endometriosis also triggers continuous recruitment of monocytes to the peritoneal cavity and leads to heightened monocyte input into the LpM pool. Using genetic and pharmacological depletion strategies we demonstrated a “proendometriosis” role for endometrial macrophages and an “antiendometriosis” role for monocyte-derived LpM (15). Although the complexity of macrophage origin and function in endometriosis is now partially revealed, the true complexity of macrophage phenotype, and how disease-modified macrophages differ from macrophages in steady-state tissues is currently unknown. The identification of unique markers that discriminate proendometriosis macrophages, will facilitate a targeted therapeutic approach to eliminate or alter disease-promoting macrophages, while leaving those critical for normal physiological tissue function intact. In the current study, we have used single-cell transcriptomics (scRNA-Seq) to investigate the transcriptional heterogeneity of both lesion-resident and associated peritoneal macrophages, and to identify population-specific markers. We have further explored the role of specific phenotypes using in vitro and in vivo functional studies, shedding new light on the role of macrophages in the pathophysiology of endometriosis. Additionally, we performed cross-species mapping of human and mouse datasets to determine the concordance of populations between the species. Taken together, we describe critical background knowledge required for the development of future targeted immunotherapy for endometriosis.

## Results

### Endometriosis-Associated Macrophages Exhibit Phenotypic Heterogeneity and Unique Markers.

**Fig. 1.**
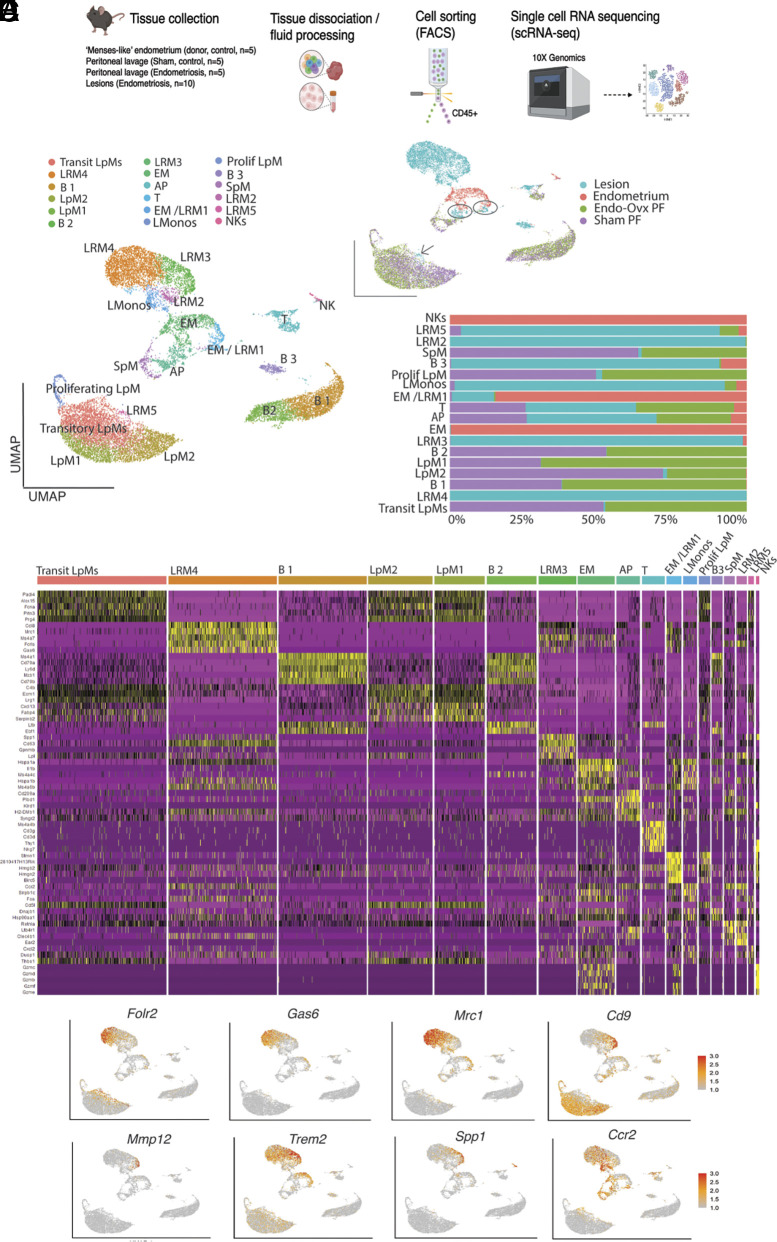
Endometriosis-associated macrophages exhibit significant transcriptomic heterogeneity. (*A*) Schematic of workflow and tissues, fluid evaluated. (*B*) UMAP projection of CD45+ cells isolated from menses-like endometrium (donor mice; n = 5), peritoneal lavage (PF) from sham mice (n = 5), PF (n = 5), and lesions (n = 10 mice) isolated from mice with endometriosis. The *Inset* is UMAP projection based on library ID. AP; antigen-presenting cells, EM; endometrial macrophages; LpM; large peritoneal macrophages, LRM; lesion resident macrophages, SpM; small peritoneal macrophages, LMonos; lesion resident monocytes, NK; natural killer cells. (*C*) Bar chart depicting cluster membership of each sample type. (*D*) Heatmap of top five differentially expressed genes (DEGs) for each cluster. (*E*) Feature plot of marker genes exhibiting restricted expression in SAM-like and TAM-like lesion-resident macrophages.

To further understand the dynamics of endometriosis-associated macrophage subpopulations and to identify phenotypic heterogeneity we performed 10X scRNA-Seq on isolated leukocytes from samples recovered from a mouse model of experimental endometriosis (12). In brief, endometriosis was induced in wild-type C57BL/6 recipient mice (recipients were ovariectomized and supplemented with estradiol) by injecting menses like donor endometrium (endometrium is exposed to hormonal manipulation, a stimulus to induce decidualization, followed by progesterone withdrawal to mirror shedding that occurs at menstruation) into the peritoneal cavity and allowing lesions to establish over 2 wk (Endo-Ovx model). We isolated CD45+ leukocytes from lesions and the peritoneal lavage of mice with endometriosis using FACS. For comparison, we also isolated leukocytes from sham mice (mice were ovariectomized, supplemented with estradiol, and subject to an injection of intraperitoneal saline instead of endometrial tissue) and eutopic “menses”-like endometrium (4 h post progesterone withdrawal) from “donor” mice. An aggregate analysis of CD45+ peritoneal lavage cells, menses endometrial tissue and endometriosis lesions was performed (Fig. 1*A*). UMAP projection revealed 18 clusters (Fig. 1*B*). We ascertained cell identity of each cluster using canonical markers (*SI Appendix*, Fig. S1); 13 of these were monocytes/macrophages/DCs based on expression of *Ccr2*, *Adgre1, Cd209a,* or *H2-Aa* expression. Other clusters were B cells, T cells, and NKs. UMAP projection based on sample ID revealed that a proportion of lesion-resident macrophages clustered with LpM (arrow) and endometrial macrophages (rings; Fig. 1 *B*, *Inset*) highlighting our previous observations that lesion-resident macrophages have different origins (15).

Endometrial-derived macrophages separated into three clusters, two of which were shared by lesion-resident macrophages. The most abundant endometrial cluster (EM) was characterized by the differentially expressed genes (DEGs) *Il1b*, *Ly6c2*, *Ms4a4c*, *S100a9,* and *S100a8* (Fig. 1*D*; see Dataset S1 for gene list). Expression of *Ly6c2* and *Ms4a4c* indicates that this population was composed predominantly of monocytes. The second cluster (AP) containing endometrial cells was characterized by the DEGs *Cd209a*, *Slamf7, Cd74,* and *Lsp1,* suggesting a mixed population of antigen-presenting cells (including DC) and was composed of endometrial, peritoneal, and lesion-resident cells. The final EM/LRM1 was characterized by expression of components of the cell cycle pathway highlighting that proliferating macrophages are present in both “menses-like” endometrium and lesions.

Peritoneal macrophages separated into five clusters comprising one cluster of SpM (*H2-Aa*, *Ccr2*, *Retnla*) and four clusters of LpM (LpM1, LpM2, Proliferating LpM, and Transitory LpM). Proliferating LpM were identified based on their expression of cell cycle/proliferative markers such as *Top2a, Cdk1, Mcm5,* and *Mki67* and were represented equally in both Endo-Ovx and Sham samples (Fig. 1*C*). Both Endo-Ovx and Sham mice exhibited a predominant population of “transitory” LpM (Fig. 1 *B* and *C*; see also *SI Appendix*, Fig. S2) which were characterized by a downregulation of *Mrc1*, *Ccr2,* and *H2-Aa* and the emergence of LpM markers including *Icam2.* In support of these cells being transitory, GO and KEGG analysis (Dataset S2) did not return any unique terms for this cluster, with many terms primarily related to translation; in line with macrophages responding to their environment and transitioning through functional states. The presence of a large population of transitory LpM corroborates data in previous studies indicating that surgery to remove the ovaries (with or without estradiol supplementation) significantly impacts the peritoneal immune environment in female mice resulting in increased macrophage replenishment (6). To begin to differentiate between embryo-derived/long-lived tissue-resident LpM and monocyte-derived LpM we initially evaluated the presence of *Ccr2* (marker of recently recruited monocytes/monocyte-derived macrophages) and *Timd4* (long-lived tissue-resident macrophages). *Ccr2* was most abundant on SpM, with expression on some transitory LpM. *Timd4* was most prominent on LpM1 and LpM2 with some sporadic *Timd4*+ cells in the transitory population (*SI Appendix*, Fig. S2*D*). LpM 1 were characterized by DEGs shared by steady-state prototypical LpM: *Cxcl13, Prg4, Alox15*, and *Fabp4* and the emergence of *Tgfb* (6). Evaluation of cluster occupancy revealed shifts in LpM populations; prototypical LpM (LpM1) were expanded, while LpM2 (characterized by *Fcna, C4b, Ecm1,* and *Lrg1* mRNAs) were depleted in Endo-Ovx mice (Fig. 1 *B* and *C*).

Lesion-resident macrophages separated into an additional four clusters. LMono expressed *Ccr2* and *Ly6c* as well as *Il1b,* indicating the cluster is comprised predominantly of monocytes. LRM2 DEGs were those traditionally associated with monocyte-to-macrophage transition and monocyte-derived inflammatory cells such as *Ccr2*, *Ear2,* and *Clec4b1* (16). Unique GO terms included those associated with “dendrite morphogenesis” and “regulation of synapse activity”, the KEGG pathways returned were “prion disease”, “Alzheimer’s disease”, and “pathways of neurodegeneration” (Dataset S2) indicating that this subpopulation may be associated with pathways of neurogenesis and neuroinflammation and supports studies (including our own), that endometriosis-associated macrophages are implicated in pathogenic recruitment and activation of nerves (13, 14). Other GO terms included “negative regulation of lymphocyte differentiation”, “negative regulation of T cell differentiation”, and “negative regulation of leukocyte differentiation”. These results indicate a role for this population in the suppression of specific immune cell differentiation pathways as a mechanism to modulate immune response in the endometriotic environment, perhaps by contributing to immunotolerance of lesions. LRM3 exhibited a transcriptional profile shared with scar-associated macrophages (SAMs)/fibrosis-associated macrophages (17) characterized by the profibrogenic genes *Spp1* and *Lgals* as well as *Trem2*, *Mmp12*, *Cd9,* and *Gpnmb.* Using feature plots, genes exhibiting cluster-restricted expression were visualized (Fig. 1*D*). *Mmp12* and *Spp1* exhibited expression specific to SAM-like cells, whereas *Cd9* and *Trem2* mRNAs were most abundant in SAM-like cells but also exhibited expression, albeit at lower levels, in some other clusters. Unique terms returned for LRM3 included “ensheathment of neurons”, “ensheathment of axons”, “neuron migration” as well as “CD4-positive, alpha-beta T cell activation” and “negative regulation of tumor necrosis factor production”, indicating that this subpopulation is involved with neuron migration, promoting T-cell response, and limiting production of proinflammatory cytokines, all supporting these macrophages as exhibiting a prorepair phenotype. Of the KEGG pathways returned, “proteoglycans in cancer” was of particular interest given the function of proteoglycans as key components of extracellular matrix (ECM) that influence cell migration, invasion, and angiogenesis (18) and indicates that these macrophages may play an important role in ECM formation, remodeling, and fibrosis. The most abundant lesion-resident population was LRM4 which was characterized by expression of *Ccl8*, *Mrc1*, *Gas6*, *Marks, Cbr,* and *Folr2,* a signature shared with many tumor-associated macrophages (TAM) (19–21). Other interesting genes that were differentially expressed in this population included *Sepp1*, *Nrp1,* and *Igf1*. Visualization using feature plots demonstrated that *Gas6* and *Folr2* exhibited predominantly restricted expression to TAM-like cells, whereas *Mrc1* exhibited lower expression in some other clusters (Fig. 1*E*). GO terms returned for TAM-like macrophages included “central nervous system development”, “positive regulation of nervous system development”, “mesenchymal cell differentiation”, “fibroblast migration”, “vasculogenesis”, and “response to ischemia” consistent with a role for these macrophages in neuroangiogenesis, tissue remodeling and repair, and response to hypoxic conditions. KEGG pathways also included “neuroactive ligand–receptor interaction”. Finally, LRM5 clustered with peritoneal macrophage populations and likely represents peritoneal macrophages that have recently infiltrated into lesions. LRM5 shared several genes with LpM such as *Prg4* and *Saa3* (Fig. 1*D*). Unique GO terms included “response to pain”, “response to fibroblast growth factor”, “keratinocyte proliferation”, and “smooth muscle cell differentiation” highlighting potential roles in tissue repair and remodeling, cellular crosstalk, and pain modulation. *Ccr2* was also visualized using a feature plot (Fig. 1*D*) and exhibited highest expression in LMonos and LRM2 with lower expression extending out into LRM4, EM, and AP.

### SAM-Like and TAM-Like Cells Arise from Monocyte Precursors and Not Infiltrating Peritoneal Macrophages.

**Fig. 2.**
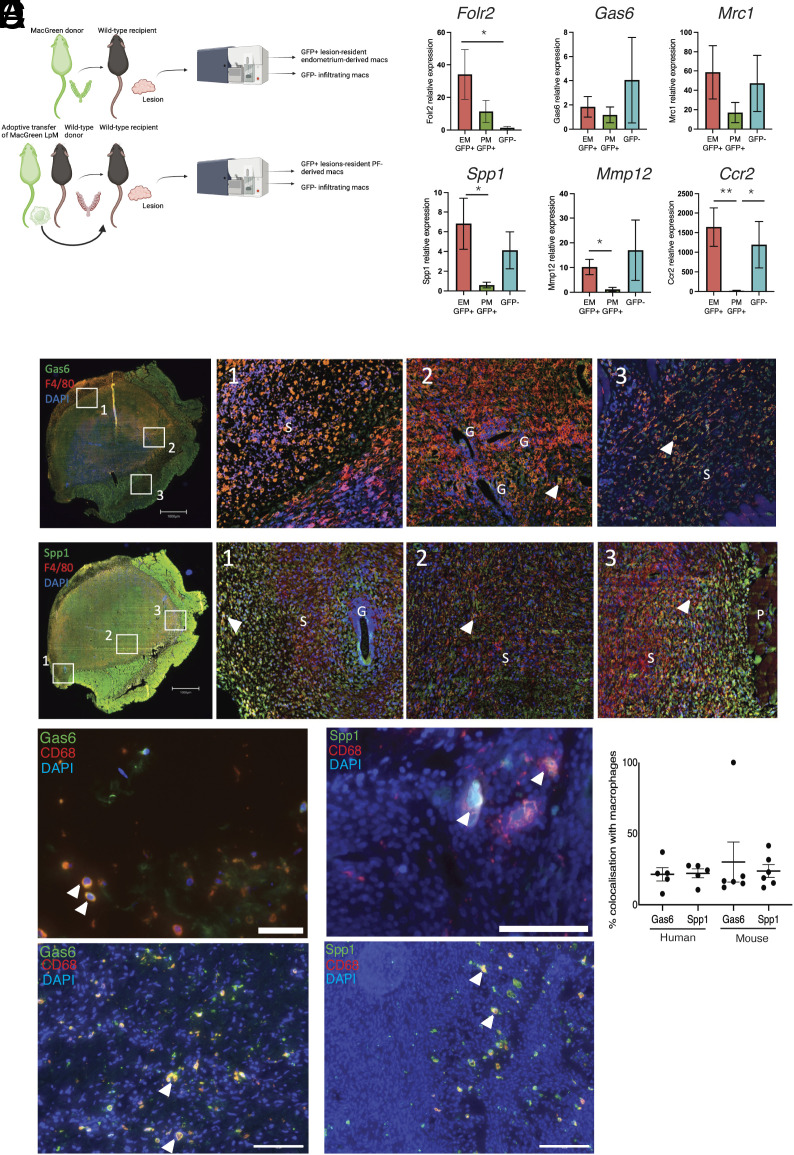
SAM-like and TAM-like cells appear to arise from monocyte precursors and not infiltrating peritoneal macrophages. (*A*) Schematic representation of fate-map experiments that enable FACs isolation of macrophages from different locations. To isolate endometrial-derived macrophages from lesions (n = 6 mice), menses-like endometrium from MacGreen donor mice was transferred into the peritoneal cavity of wild-type recipients and lesions allowed to develop for 14 d. Lesions were recovered and GFP+ endometrial-derived macrophages were isolated. The GFP− macrophage fraction was constituted from monocytes that extravasate from lesion blood vessels and differentiate into macrophages and infiltrating peritoneal macrophages (n = 6). To isolate peritoneal macrophages from lesions we performed adoptive transfer of LpM (F4/80+) isolated from the peritoneal lavage of MacGreen mice and injected into the peritoneal cavity of wild-type recipient mice at the same time as endometrium from wild-type mice. Lesions formed over 14 d, followed by FACs isolation of GFP+ (peritoneal) lesion-resident macrophages (n = 6). The GFP− fraction was also collected and was constituted by endometrial-derived macrophages and extravasated monocyte-derived macrophages (n = 6, collectively n = 12 for the GFP− fraction). (*B*) Relative mRNA concentrations of TAM-like (*Folr2*, *Gas6,* and *Mrc1)* and SAM-like (*Spp1* and *Mmp12*) macrophage markers assessed by QPCR. Data presented are mean ± SEM. (*C*) Immunolocalization of Gas6 and Spp1 (green) and colocalization with F4/80 (red) in mouse lesions (G; gland, S; stroma, P; peritoneum). (*D* and *E*) Immunolocalization of Gas6 and Spp1 (green) and colocalization with CD68 (red; arrow heads denote cells exhibiting colocalization) in lesions recovered from women with endometriosis (D: peritoneal lesions, E: endometrioma). (Scale bar, 100 μM.) (*F*) Quantification of colocalization in human peritoneal lesions (n = 5) as well as mouse lesions (n = 6). Data presented are mean ± SEM. Statistical analysis was performed using a Kruskal–Wallis and a Dunn’s multiple comparison test. *P < 0.05, **P < 0.01.

We previously determined that macrophages in endometriosis lesions have different origins (15). In this study, we sought to align lesion-resident macrophage origin to the different phenotypes uncovered using single-cell discovery. To achieve this, we performed reciprocal transfer and adoptive transfer experiments using cells derived from MacGreen (Csf1r-EGFP) mice (Fig. 2*A*) to facilitated isolation of ontogenetically distinct populations from lesions followed by qPCR for TAM-like and SAM-like markers. To isolate endometrial-derived monocytes/macrophages from endometriosis lesions we injected menses-like endometrium from MacGreen donors into the peritoneal cavity of wild-type recipients. At 2-wk post tissue-injection lesions were collected, digested, and FACS sorted into GFP+ (endometrial-derived) and GFP- (recipient-derived) monocytes/macrophages. In a separate experiment, we induced endometriosis using wild-type donor endometrium and wild-type recipients, and simultaneously performed adoptive transfer of FACS-sorted GFP+ LpM (collected from a naïve mouse) into the cavity of recipient mice. As before, lesions were collected and sorted into GFP+ (lesion-resident LpM) and GFP− monocytes/macrophages. Separately, we also identified that both Tim4+ and Tim4− LpM trafficked into lesions (*SI Appendix*, Fig. S3). mRNA concentrations of TAM-like and SAM-like markers were evaluated. The TAM-like marker *Folr2* exhibited highest mRNA concentrations in lesion-resident macrophages derived from the endometrium (*P* < 0.05; Fig. 2*B*)*. Gas6* mRNA was most abundant in cells derived from infiltrating monocytes*. Mrc1* exhibited higher expression in lesion-resident macrophages derived from the endometrium and infiltrating monocytes (Fig. 2*B*). The SAM-like markers *Spp1* (*P* < 0.05) and *Mmp12* (*P* < 0.05) exhibited increased expression in endometrial-derived macrophages compared to peritoneal derived macrophages. *Ccr2* mRNA concentrations were also elevated in both endometrial-derived lesion-resident macrophages (*P* < 0.01) and the GFP- host-derived population (*P* < 0.05) compared to peritoneal-derived lesion-resident macrophages. Thus, the data are consistent with donor endometrial macrophages and GFP- infiltrating populations being predominantly monocyte-derived as inferred by the scRNA-Seq data presented above [EM and LMono clusters and Cousins et al. (22)]. Our data indicate that monocyte-derived cells appear to give rise to prodisease TAM and SAM-like cells. Conversely, the lesion-resident macrophages that are derived from LpM (15) possess a mature macrophage phenotype, and the qPCR data are consistent with these cells having limited differentiation capacity within the lesion. Using immunofluorescence, TAM-like (LRM4; Gas6) and SAM-like (LRM3; Spp1) macrophages were detected in situ in mouse (Fig. 2*C*) and human (Fig. 2 *D* and *E*) lesions, and no significant differences were detected in the abundance of each population (Fig. 2*F*).

### Lesion-Resident Folr2+ Macrophages Exhibit “prodisease” Properties.

To begin to ascertain whether the TAM-like macrophages identified in the scRNA-Seq dataset exhibited prodisease properties in line with their transcriptional phenotype, we FACS sorted Folr2+ macrophages from the peritoneal lavage and lesions of mice with experimentally induced endometriosis (*SI Appendix*, Fig. S4*A*). A greater proportion of lesion-resident macrophages were Folr2+ compared to peritoneal macrophages (*SI Appendix*, Fig. S4*B*). Isolated Folr2+ and Folr2− macrophages were cultured in vitro to obtain conditioned media (CM) which was then used in downstream functional assays. Initially, we exposed human endometrial stromal cells to macrophage CM for 3 d followed by qPCR on extracted RNA. Macrophage CM had no impact on the expression of the proliferation marker *Mki67*, whereas CM from lesion-resident Folr2+ macrophages (but not lesion-resident Folr2− or peritoneal macrophages) induced elevated mRNA concentrations of *Col1a1* and *Tgfb1* (*P* < 0.05; *SI Appendix*, Fig. S4*C*). This was consistent with pathophysiological processes taking place within lesions such as extracellular matrix deposition/fibrosis and transdifferentiation. Next, human umbilical vein endothelial cells (HUVECs) were plated on Matrigel® in transwell plates and exposed to macrophage CM. Multiple parameters were evaluated including branches, segments, junctions, and meshes (*SI Appendix*, Fig. S4*D*). We found that CM from lesion-resident Folr2+ macrophages induced a significant and rapid increase in angiogenesis (meshes) at 6 h (*SI Appendix*, Fig. S4*E*, *P* < 0.01), whereas CM from lesion-resident Folr2− macrophages did not. Both Folr2+ and Folr2− peritoneal macrophages induced a significant increase in angiogenesis (meshes) at 8 h and 6 h, respectively.

### A Unique Population of Monocyte-Derived LpM is Evident in Mice with Experimental Endometriosis.

**Fig. 3.**
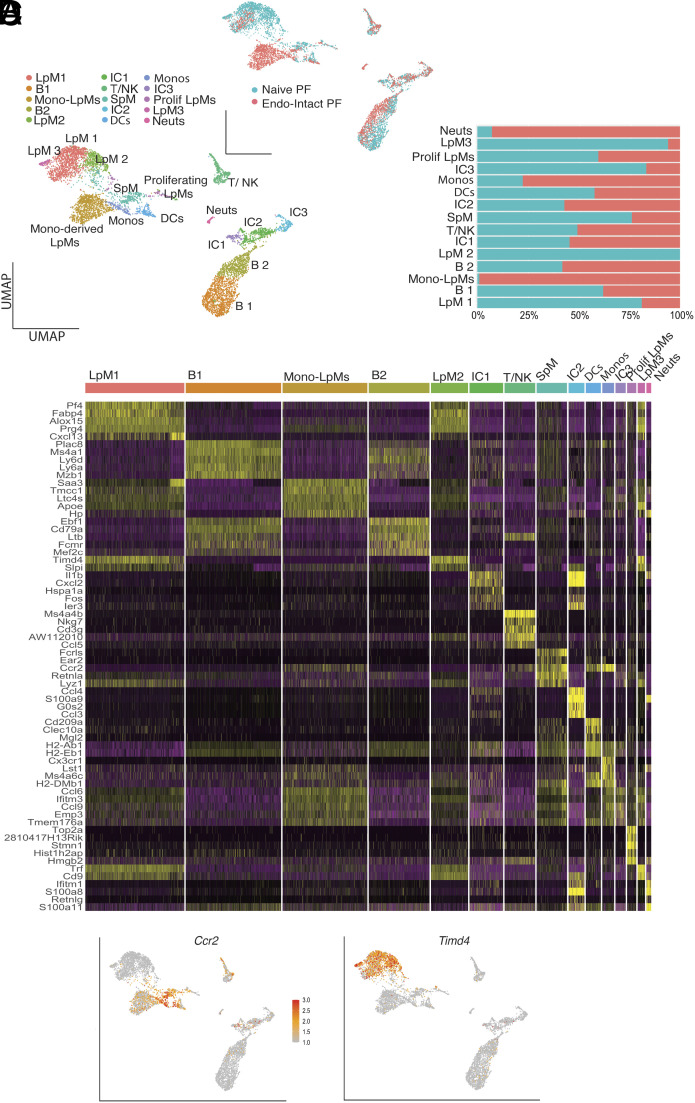
A unique population of monocyte-derived LpM is evident in mice with experimental endometriosis. (*A*) UMAP projection of CD45+ cells isolated from the peritoneal lavage from naïve mice (Naïve PF; n = 5) and mice with endometriosis (ovaries intact; Endo-Intact PF; n = 5). The *Inset* shows UMAP based on library ID. (*B*) Bar chart showing cluster membership of different samples. (*C*) Heatmap showing top five DEGs per cluster. (*D*) Feature plots of *Ccr2* and *Timd4* expression.

Our previous work suggested that monocyte-derived LpM confer protection against development of endometriosis lesions. In experiments that limited recruitment of monocytes to the peritoneal cavity, significantly more lesions developed, whereas reprogramming the cavity such that embryo-derived LpM were replaced by monocyte-derived LpM resulted in significantly fewer lesions (15). However, in our scRNA-Seq studies, the altered immune environment induced by surgery (abundant transient LpM in both Endo-Ovx and Sham; Fig. 1 and *SI Appendix*, Fig. S2) decreased the distinction with which transcriptional alterations in peritoneal macrophages could be attributed to endometriosis. Thus, we repeated the experiment using ovary-intact mice (Endo-Intact and Naïve). UMAP projection revealed 15 clusters and cell identity was assigned based on expression of canonical markers (*SI Appendix*, Fig. S5). In Endo-Intact mice, an almost entirely unique population of monocyte-derived LpM was evident (Fig. 3 *A* and *B*) and characterized by the presence of *Ccr2* and loss of *Timd4* (Fig. 3*D*). Identification of this population using scRNA-Seq further substantiates our previous findings that endometriosis triggers monocyte recruitment and heightened monocyte input into the LpM pool (15), and builds on these data to indicate that monocyte-derived LpM are transcriptionally unique. Monocyte-derived LpM were characterized by DEGs *Saa3*, *Apoe*, *Pid1*, *Ltc4s*, *Cebpb,* and *Ccl6* (Dataset S3 for gene list). Unique GO terms for this cluster included “positive regulation of cellular process”, “cellular response to stimuli”, and “regulation of metabolic process”. Unique KEGG terms included “lipid and atherosclerosis”, “sphingolipid signaling pathway”, “endocytosis”, and “estrogen signaling pathway” (Dataset S4). This population shares several DEGs with the transitory LpM population in the Ovx dataset, and *Apoe* is a consistent DEG across transitory LpM, LpM2 (Ovx dataset; Fig. 1 and *SI Appendix*, Fig. S2), and monocyte-derived LpM (Intact dataset). LpM1 exhibits a comparable prototypical transcriptional phenotype in the two datasets, whereas LpM3 (Intact dataset) clusters separately from LpM1 but shares many of the same tissue-resident markers.

### Apoe Regulates Peritoneal Macrophage Populations and Limits Growth of Lesions in Experimental Endometriosis.

**Fig. 4.**
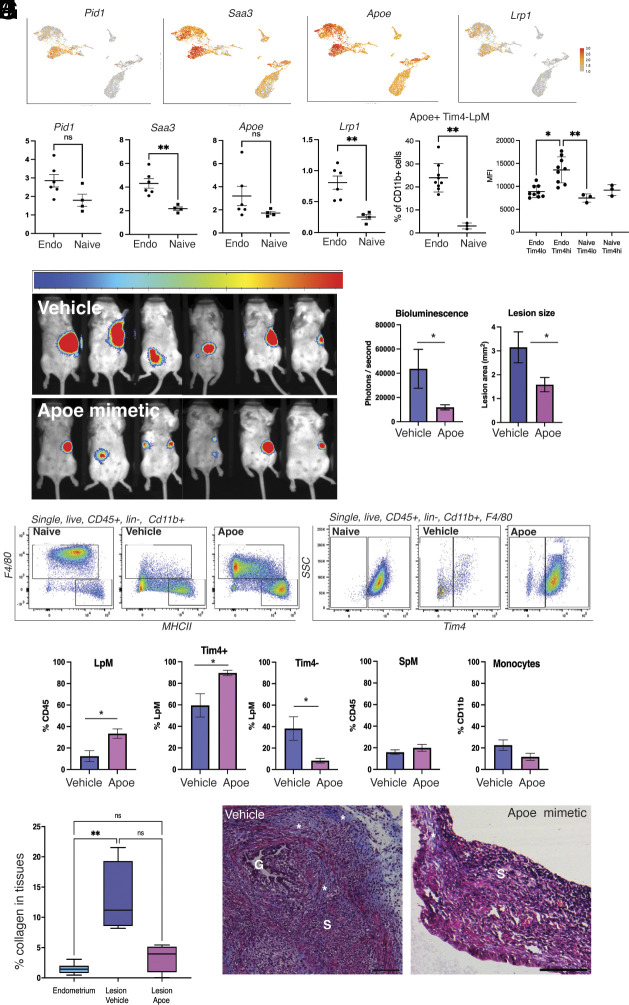
Apoe regulates peritoneal macrophage populations and limits growth of lesions in experimental endometriosis. (*A*) Feature plots of monocyte-derived LpM subpopulation marker genes, *Pid1*, *Saa3*, *Apoe,* and *Lrp1*. (*B*) QPCR for marker genes on FACs-sorted Tim4− (monocyte-derived) LpM isolated from naïve mice (n = 4) and mice with experimental endometriosis (n = 6). Data presented are mean ± SEM. (*C*) Quantification of Apoe+, Tim4− LpM in mice with endometriosis (Endo; n = 9) and those without (Naïve; n = 3) analyzed via flow cytometry. (*D*) Mean fluorescence intensity of Apoe on different macrophage populations in mice with (Endo) and without (naïve) endometriosis. Data presented are mean ± SD. Mice with induced endometriosis were injected i.p. with and the Apoe mimetic peptide COG 133 (Generon A1131; 300 μM in 200 μL dH_2_O, n = 6) or vehicle (n = 6) daily for 14 d from day of endometriosis induction. Bioluminescent imaging of lesions was performed twice weekly, and the images shown are from day 13 of the experiment (*E*). (*F*) Quantification of bioluminescent signal on day 13 and quantification of lesion area following histological processing, image capture, and measurement using Fiji. (*G*) Representative flow plots of peritoneal lavage showing LpMs (F4/80^hi,^ MHCII^lo^) and SpM (F4/80^lo^, MHCII^hi^; *Left* side) and Tim4+ LpMs (*Right* side) in naïve and endometriosis mice injected with vehicle or Apoe mimetic. (*H*) Quantification of peritoneal macrophages and monocytes (flow data). Data presented are mean ± SEM. (*I*) Masson Trichrome stain was performed on lesions collected from endometriosis injected with either vehicle or Apoe mimetic and areas of collagen deposition (blue stain) quantified. Data (n = 6) are presented as a box plot with the min and max. (*J*) Representative Masson Trichrome images of lesions. G; glands, S; stroma, asterisks; areas of ECM accumulation. Statistical significance was ascertained using a Student’s *t* test or Kruskal–Wallis with Dunn’s multiple comparison test. *P < 0.05, **P < 0.01.

Initially, we presumed that the protective mechanism of monocyte-derived LpM must be a result of increased phagocytic capability. However, in FACS-sorted monocyte-derived LpM vs. prototypical LpM (Tim4− vs. Tim4+) isolated from Endo-Intact and naïve mice, we found no significant differences in phagocytosis (*SI Appendix*, Fig. S6). *Pid1*, *Saa3, Apoe,* and *Lrp1* were key DEGs in the monocyte-derived LpM population (Fig. 4*A*), and qPCR analysis also confirmed an upregulation of these genes in Tim4− peritoneal macrophages isolated from mice with induced endometriosis compared to those without (*Saa3*, *Lrp1,* *P* < 0.01; Fig. 4*B*). Given the association of these genes with lipid processing, we evaluated the uptake of lipid by FACS-sorted Tim4+ and Tim4− LpM derived from mice with (n = 9) and without (n = 3) endometriosis. We found no significant difference in lipid uptake between the macrophage populations (*SI Appendix*, Fig. S7*A*). Of the key genes, *Apoe* appeared to have an implicit role in LpM function in endometriosis; Apoe+ Tim4− LpM were significantly more abundant in mice with endometriosis compared to those without (Fig. 4*C*; *P* < 0.01), albeit the mean fluorescence intensity was higher in Tim4+ LpM derived from mice with endometriosis (Fig. 4*D*). Given that monocyte-derived LpM have been demonstrated to rapidly gain expression of Tim4 in the presence endometriosis lesions (23) we propose that the Tim4+, Apoe+ LpM represent a more mature phenotype (“endometriosis educated”) derived from Tim4− monocyte-derived LpM and that they express higher levels of Apoe. Collectively, Apoe+ LpM are significantly more abundant in mice with endometriosis (*SI Appendix*, Fig. S7 *B* and *C*; *P* < 0.05). Next, we aimed to perform a gain of function experiment whereby exogenous Apoe (mimetic) was delivered into the peritoneal cavity of mice with experimentally induced endometriosis (intact mice); the Apoe mimetic (COG-133, 3 μM in 200 μL sterile H_2_O) or vehicle were delivered daily (via intraperitoneal injection), with injections initiated at the same time as ectopic tissue transfer and for 2 wk following. Lesions were monitored using noninvasive bioluminescent imaging (Fig. 4*E*). Treatment with the Apoe mimetic significantly reduced the bioluminescent signal (Fig. 4*F*; *P* < 0.05) and cross-sectional area (*P* < 0.05) of lesions. Flow cytometry revealed a loss of LpM in endometriosis mice exposed to daily i.p. injections of vehicle, consistent with the expected macrophage disappearance reaction. Conversely, mice exposed to the Apoe mimetic exhibited significantly more Tim4+ LpM (Fig. 4 *G* and *H*; *P* < 0.05). Numbers of recruited SpM were similar in both Apoe and vehicle-treated mice. In pulmonary fibrosis, Apoe produced by monocyte-derived macrophages plays a key role in the resolution of established lesions in the lung. In this context, Apoe was found to promote phagocytosis of type 1 collagen by macrophages in an LRP-dependent manner (24). Thus, we used Masson Trichrome staining to evaluate the amount of collagen deposition in lesions collected from vehicle-treated mice with endometriosis, as well as those injected with the Apoe mimetic. Compared to the eutopic endometrium, lesions collected from vehicle-treated mice exhibited significantly greater levels of collagen (*P* < 0.01), whereas there was no significant difference in collagen content between eutopic endometrium and lesions from Apoe treated mice (Fig. 4 *I* and *J*).

### Cross-Species Comparison Reveals Remarkable Concordance in Endometriosis-Associated Macrophage Phenotypes and Conserved Population Markers.

**Fig. 5.**
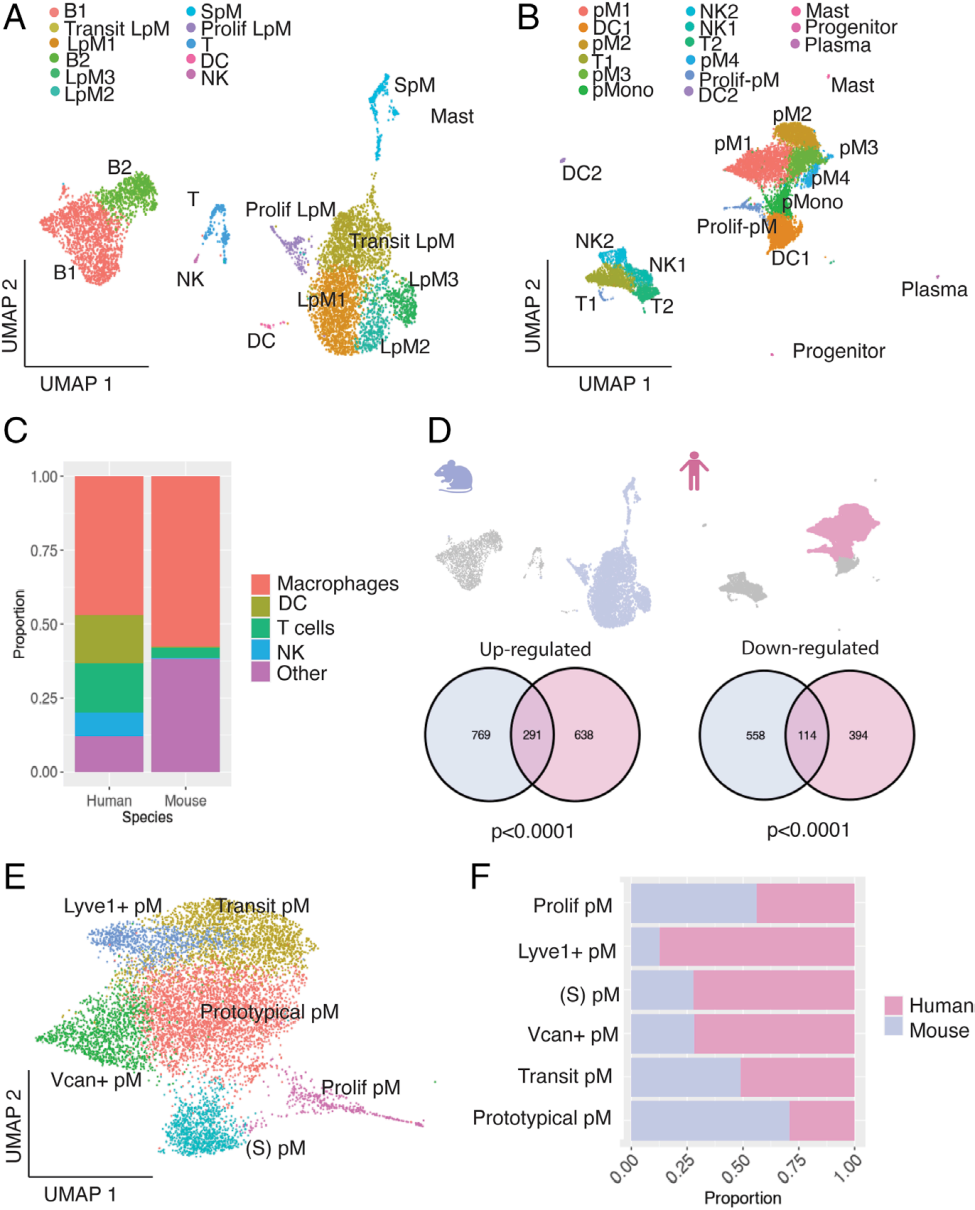
Cross-species mapping of mouse and human peritoneal macrophages. (*A*) UMAP projection of CD45+ cell derived from mouse peritoneal fluid (Ovx-Endo; Seurat v.5), (*B*) UMAP projection of CD45+ cells derived from human peritoneal fluid (patient with endo only; Zou et al. publicly available dataset). (*C*) Bar chart showing the proportions of each cell type present in the two datasets. The “other” population includes other cells excluding macrophages, DC, T, and NK (e.g., B cells, mast cells). (*D*) The macrophage subset was extracted from each dataset (lilac and pink for mouse and human, respectively) and evaluated for shared up- and down-regulated genes; see Venn diagrams. (*E*) Cross-species integration of single-cell RNA-sequencing data was performed to map mouse and human macrophage subpopulations. (*F*) Bar chart showing cluster member proportions for each species.

Design and testing of future macrophage-targeted therapies for endometriosis relies on the understanding of how macrophage subpopulations are similar/unique between the species. Thus, we performed a comparative single-cell transcriptomic analysis of mouse endometriosis-associated macrophages from our preclinical model and their human counterparts. Individual analyses of mouse peritoneal lavage and lesion-resident CD45+ cells from the Endo-Ovx dataset were comparatively analyzed with human CD45+ cells from an endometriosis peritoneal fluid dataset (n = 1) (25) and an aggregation of nine peritoneal endometriotic lesion samples (26), respectively. After the initial quality control steps and CD45+ filtering, we isolated single-cell transcriptomes from 6,026 mouse and 8,384 human peritoneal fluid cells. UMAP projection revealed 11 clusters of cells derived from Endo-Ovx mouse peritoneal lavage, with macrophages being the predominant immune cell type (Fig. 5 *A*–*C*). Cell populations aligned with our previous aggregate analysis (Fig. 1 and *SI Appendix*, Fig. S2) and we detected a new cluster, LpM3, that was not originally identified in this dataset and was characterized by expression of complement receptors and lysozyme 2 (*C1qa, C1qc,* and *Lyz2*). In the human dataset, UMAP projection revealed 15 clusters, concordant with the analysis by Zou et al. (25), with similarly predominant macrophages (Fig. 5 *B* and *C*). Initially, MAST analysis was used to identify exact-match DEGs expressed in homologous combined macrophage clusters, with human DEGs converted to mouse orthologs and both filtered by direction of change. Hypergeometric testing revealed a significant overlap between exact-match up-regulated DEGs in the mouse and human peritoneal fluid macrophages, with an 8.5-fold greater enrichment in the overlap than would be expected by chance (hypergeometric *P* < 0.0001), and a 9.7-fold enrichment in the overlap in down-regulated DEGs (hypergeometric *P* < 0.0001) (Fig. 5*D*). Next, we performed an integrated cross-species analysis of the peritoneal fluid datasets. Following cross-species homology mapping, monocytes/macrophages were extracted from the datasets for integration. UMAP projection of integrated peritoneal fluid datasets composed of 3,644 mouse and 5,113 human cells revealed 6 populations of monocytes/macrophages (Fig. 5*E*), with UMAP projection by species showing high concordance between datasets (*SI Appendix*, Fig. S8*A*). Cluster validation was performed by mapping cell barcodes from specific populations, identified in individual species comparative analysis, onto the integrated UMAP (*SI Appendix*, Fig. S8 *C* and *D*). Visualization of clusters (*SI Appendix*, Fig. S8*E*), revealed *Ccr2* to be up-regulated on cells that are analogous with mouse SpM [named (S) pM in the integrated dataset] and Vcan+ pM, indicating recent differentiation from monocytes, with highest expression on (S) pM alongside the characteristic high *H2-Aa* and low *Adgre1* expression seen in mouse SpM. *Ccr2* was expressed at a lower level in Vcan+ pM, alongside expression of *S100a8* and *S100a9* (Dataset S5 for gene list). *Timd4* was up-regulated on prototypical pM and Lyve1+ pM, indicative of long residence. Prototypical pM expressed high *Adgre1* and genes characteristic of steady-state prototypical LpM in mice: *Cxcl13, Prg4, Alox15, and Fabp4.* Lyve1+ pM also expressed scavenger receptors *Marco*, *Cd163,* and complement *C2*. Transitory (Transit) pM also expressed *Lyve1*, although at a lower level, alongside downregulation of *H2-Aa* and *Ccr2*. As previously shown in Fig. 1*A*, a proliferative population (Prolif pM) was identified (*Top2a, Cdk1,* and *Mki67).* Evaluation of cluster occupancy by species revealed proliferative and transitory populations to be consistent; however, Lyve1+ pM and prototypical pM populations were expanded in humans and mice, respectively (Fig. 5*F*). Of all the clusters, the Lyve+ pM population was significantly underrepresented in the mouse dataset suggesting this population is largely unique to the human. Unique GO terms for this cluster included “neurogenesis”, “neuron projection development”, and “neuron development”; others included “negative regulation of cellular metabolic process”, “phosphorylation”, and “regulation of intracellular signal transduction”. Unique KEGG terms included endocytosis, “pathways in cancer”, and “lipid and atherosclerosis” and “growth hormone synthesis and secretion” among others (Dataset S6). As Zou et al. used patients that had not been exposed to hormonal treatment for 6 mo, we also performed the cross-species mapping with peritoneal macrophages derived from the Endo-Intact model (*SI Appendix*, Fig. S9). Hypergeometric testing revealed a significant overlap between exact-match up-regulated DEGs in the mouse and human peritoneal fluid macrophages, with a 10.5-fold greater enrichment in the overlap than would be expected by chance (hypergeometric *P* < 0.0001), and a 14.1-fold enrichment in the overlap in down-regulated DEGs (hypergeometric *P* < 0.0001; *SI Appendix*, Fig. S8*D*). UMAP projection of integrated peritoneal fluid datasets composed of 1,275 mouse and 5,113 human cells revealed six populations of monocytes/macrophages (*SI Appendix*, Fig. S9*E*), in line with what was observed with the Endo-Ovx dataset. Evaluation of cluster occupancy by species revealed similar proportions to the Endo-Ovx comparison, although transitory pM were evidently more expanded in the Endo-Intact mapping results and this corresponds with the abundant monocyte-derived LpM in that dataset. Lyve1+ and Vcan+ pM were less abundant in the Endo-Intact dataset. Proliferative pM, (S) pM, and prototypical pM exhibited similar proportions in both comparisons, suggesting that these populations are consistent, regardless of the hormonal/surgical status of the mouse and are the most equally represented populations in the human samples.

**Fig. 6.**
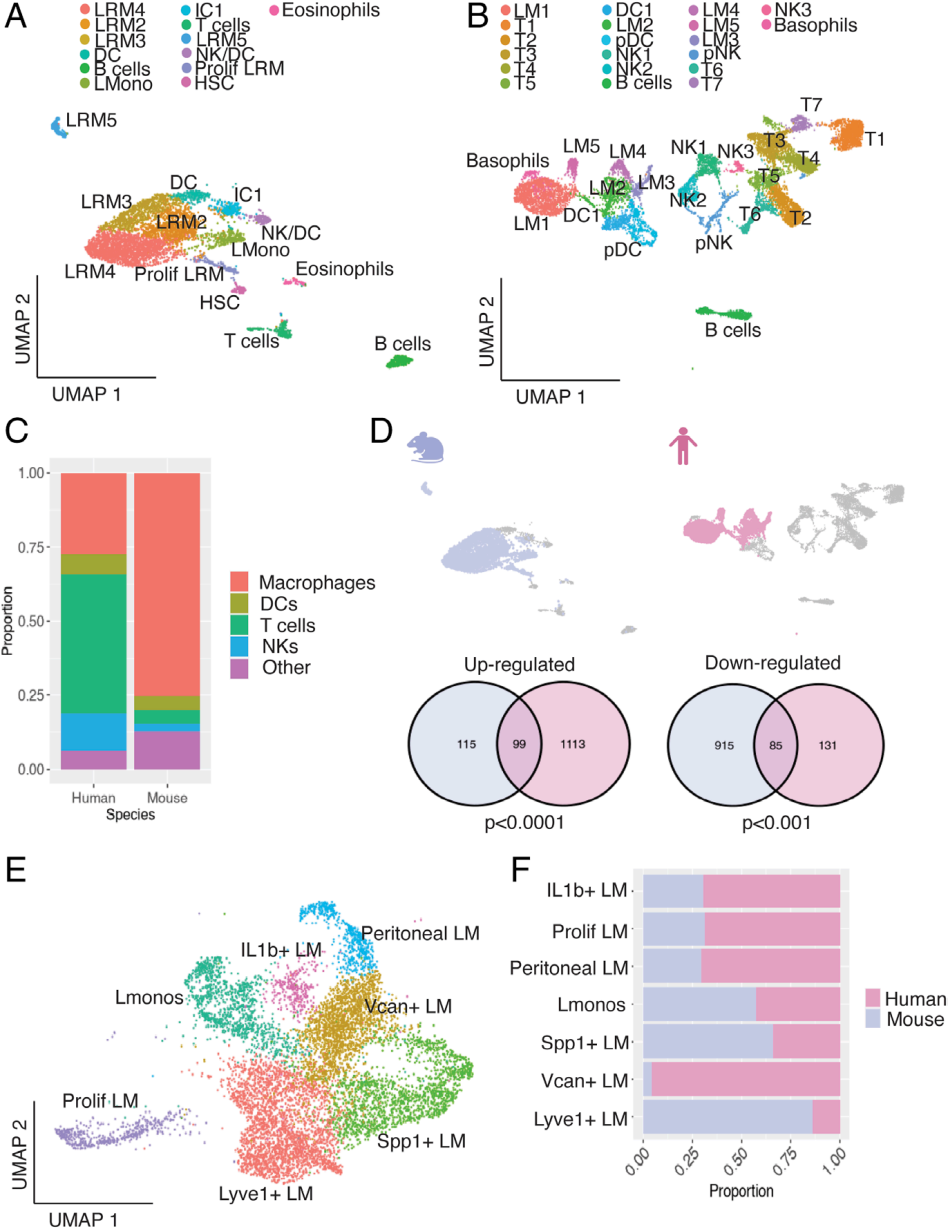
Cross-species mapping of mouse and human lesion-resident macrophages. (*A*) UMAP projection of CD45+ cells derived from lesions recovered from a mouse model of experimental endometriosis (Ovx-Endo; Seurat v.5), (*B*) UMAP projection of CD45+ cells derived from human endometriosis lesions (peritoneal only; Tan et al., publicly available dataset). (*C*) Bar chart showing the proportions of each cell type present in the two datasets. The other population includes other cells excluding macrophages, DC, T, and NK (e.g., eosinophils, basophils, B cells). (*D*) The macrophage subset was extracted from each dataset (lilac and pink for mouse and human, respectively) and evaluated for shared up- and down-regulated genes; see Venn diagrams. (*E*) Cross-species integration of single-cell RNA-sequencing data was performed to map mouse and human macrophage subpopulations. (*F*) Bar chart showing cluster member proportions for each species.

In the lesion datasets, we isolated the single-cell transcriptomes from 5,494 mouse and 11,965 human CD45+ cells. UMAP projection revealed 13 clusters in the Endo-Ovx mouse lesions, as per our previous aggregate analysis, with macrophages accounting for >75% of cells (Fig. 6 *A*–*C*). In the human endometriosis lesions, 20 clusters were identified, with macrophages far less prevalent and accounting for just over 25% of cells, concordant with prior analysis by Tan et al. (26) (Fig. 6 *B* and *C*). Comparisons of DEGs from the homologous mouse and human lesion macrophage clusters revealed an 11-fold enrichment in the overlap of common up-regulated exact-match DEGs (hypergeometric *P* < 0.0001), and a 3.7-fold enrichment in the overlap of exact-match down-regulated DEGs (hypergeometric *P* < 0.001; Fig. 6*D*). Integrated cross-species analyses of the lesion transcriptomic datasets were performed using one-to-one orthologs as described above. UMAP projection of integrated lesion datasets composed of 4,858 mouse and 5,517 human macrophages revealed seven clusters (Fig. 6*E*). Compared to peritoneal macrophages, less alignment was seen between lesion macrophages across the two species (*SI Appendix*, Fig. S10*A*). Cluster validation was performed as before (*SI Appendix*, Fig. S9*B*). *Ccr2* was found to be up-regulated on a substantial lesion-resident monocyte (Lmono) population, alongside high *H2-Aa* and *Clec4b1* (Dataset S7), indicating these cells were recently monocyte derived. Il1b+ LM expressed high levels of *Il1b,* and several down-regulated genes, suggesting a transitory population. Lyve1+ LM expressed *Lyve1, Folr2, Mrc1,* and *Gas6*, indicating a TAM-like gene signature as seen in the mouse dataset. Spp1+ LM expressed *Spp1, Lgals, Trem2,* and *Cd9,* concordant with a SAM-like gene signature. *Lgals* was also expressed by Vcan+ LM, alongside *S100a8/9* expression. Peritoneal LM highly expressed prototypical LpM-like genes, including *Prg4*, *Alox15,* and *Fabp4,* suggesting infiltration of peritoneal macrophages into lesions as previously seen in the mouse and in the human data (26). Prolif LM expressed many markers of proliferation including *Top2a* and *Mki67.* Evaluation of cluster occupancy by species revealed the SAM-like Spp1+ pM and monocytic populations were relatively consistent; however, Vcan+ pM and Lyve1+ pM populations were expanded in humans and mice, respectively (Fig. 6*F*). Further analysis of Vcan+ macrophages derived from lesions is warranted as these appear to be specific to the human. However, GO analysis only returned three unique terms: “tyrosine phosphorylation of STAT protein”, “regulation of tyrosine phosphorylation of STAT protein”, and “post transcriptional gene silencing” and no unique KEGG terms (Dataset S8). These comparative and integrated cross-species analyses highlight the phenotypic concordance between specific endometriosis-associated macrophage populations in humans and a mouse model of experimental endometriosis, while elucidating species-specific population enhancements. Taken together, these results are vital for further studies on the development of macrophage-directed therapies for the treatment of endometriosis.

## Discussion

In the current study, we performed single-cell profiling on macrophages recovered from the endometriotic niche (lesions and peritoneal cavity) of a preclinical model, revealing the full complexity of macrophage phenotype and aligning transcriptomic signatures to macrophages with prodisease and proresolving properties. Here, we shed new light on population-specific markers and reveal the concordance between human and mouse endometriosis-associated macrophages.

scRNA-Seq analysis identified seven distinct lesion-derived monocyte/macrophage clusters in the model, highlighting heterogeneity of macrophage subpopulations far beyond those previously identified (15). Some lesion-derived cells clustered with either endometrial or peritoneal macrophages, highlighting that previously identified origins can also be found at the transcriptional level. Of note, a recognizable pattern of monocyte-macrophage transition traditionally associated with wound healing could be observed in the data (27). In lesions, this cycle was characterized by a significant monocyte population likely recruited from the circulation, an early proinflammatory macrophage population (LRM2) and prorepair phenotypes associated with ECM remodeling (LRM3; SAM-like macrophages) and promotion of cell proliferation and angiogenesis (LRM4; TAM-like macrophages). It is likely that this cycle of recruitment and differentiation within lesions is continuous. We have used a single timepoint (2-wk post endometrial tissue transfer) for these studies, and thus have provided a snapshot of macrophage phenotypes in lesions. Future work, evaluating macrophage phenotype dynamics during progression/resolution of endometriosis will be important.

Endometriosis lesions and tumors have been referred to as “wounds” that do not resolve, given their propensity for characteristic inflammation, cell proliferation (regeneration), vascularization and innervation, and transdifferentiation. Tumor macrophages also exhibit transcriptomic and functional heterogeneity analogous to what we observed in endometriosis lesions and a framework has been established in order to classify the spectrum of TAM phenotypes (28). Although some key markers (*Spp1*, *Folr2*, *Trem2*) were shared between TAM (28) and LRM subpopulations, we also observed some dichotomy, so the nomenclature was less useful in this study.

Of the three identified ontogenies of lesion-resident macrophages (15), we have demonstrated that endometrial and recruited monocyte-derived macrophages appear to give rise to prodisease macrophages, while peritoneal-derived macrophages exhibited restricted differentiation capacity in lesions. Recruitment/trafficking of peritoneal macrophages during injury has been consistently observed in different visceral organs. During intestinal injury, F4/80^hi^, GATA6+ LpM accumulate at damaged sites in a Ccr2-independent manner. Once at the site of injury, peritoneal macrophages disassemble necrotic cells and contribute to revascularization and collagen deposition (29). As it was noted that the lamina propria of the intestine exhibited an accumulation of Ccr2+ monocytes, whereas peritoneal macrophages infiltrated the muscularis of the intestine which has a lower vascular density, the authors proposed this illustrated the importance of blood flow–independent macrophage recruitment for rapid tissue repair. Consistent with this, it may be presumed that peritoneal macrophages play a critical role in establishment of endometriosis lesions, prior to development of neovessels. While LpM trafficking to lesions do not appear to adopt a prodisease phenotype, GO analysis of prototypical LpM suggests roles in “cell–cell interaction” and “migration”, “platelet activation”, and “sprouting angiogenesis” consistent with a role in wound repair and aligned with a key role for prototypical LpM in repair of damaged peritoneal lining (30, 31). Thus, we suggest that LpM are vital for the first step of attaching refluxed endometrial tissue to the peritoneal lining, establishment of lesions, and development of a blood supply.

Our previous findings indicated that endometrial macrophages are prodisease (15). In this study, our scRNA-Seq data indicate that endometrial-derived “macrophages” are largely constituted of monocytes and early, actively proliferating macrophages. We also demonstrated that monocytes recruited directly to lesions eventually yield a prodisease phenotype, indicating that it is largely lesion-resident monocyte-derived macrophages that promote the growth of lesions, neuroangiogenesis, fibrosis, and ultimate maintenance of the lesion, after the initial LpM-mediated attachment phase. In cancer, the previous dogma that monocyte-derived macrophages recruited directly to the tumor exclusively gave rise to TAMs has been challenged and it is now accepted that in some cancers, embryonic-derived tissue-resident macrophages are major contributors to the TAM pool (32). In general, high TAM infiltration correlates with poor outcomes given their widely accepted role in promoting angiogenesis, tumor growth, ECM remodeling, and inhibition of antitumor responses, e.g., t-cell-mediated cytotoxicity (33–35). However, in some cancers, TAM can be associated with enhanced antitumor properties (36). Several studies have now suggested that ontogeny plays a key role in pro- vs. antitumor activity. In endometriosis, monocyte-derived macrophages appear to adopt two different prodisease phenotypes which we refer to as TAM-like or SAM-like. *Gas6* was highly abundant in the TAM-like population. By binding to its receptors (Axl, Mertk, Tyro3), Gas6 is known to significantly affect cell cycle progression in cancer cells, promote angiogenesis, and modulate the immune environment of the tumor (37). In other systems, this pathway has been intimately linked with the process of fibrosis, including in lung and liver fibrosis (38). In patients with intrauterine adhesions (IUA), CD301+ endometrial macrophages exhibited increased abundance and secreted Gas6 that promoted endometrial fibrosis (39). Ccl8 was also a top DEG in the TAM-like population and has previously been shown to promote progression of tumor cells, induce invasion and stem-like traits in glioblastoma (19), and is also negatively correlated with patient prognosis in several different cancers (40). It is also known to be induced by lactate in TAM (41), consistent with altered metabolism and increased lactate levels in both cancer (42) and endometriosis (43). TAM-like macrophages also exhibited restricted and elevated expression of Folr2, a reliable marker induced in both lesion-resident TAM-like macrophages and some peritoneal macrophages of mice with endometriosis. Folr2 expression is associated with protumor macrophages that exhibit an anti-inflammatory and immunosuppressive microenvironment in several cancers (44); however, in some indications (e.g., breast cancer), it has been associated with CD8+ T cell infiltration and better patient survival (44). In this study, isolated lesion Folr2+ macrophages exhibited a proangiogenic and profibrotic role, consistent with expression of other prodisease genes in this population. The second prodisease population identified in lesions exhibited a scar-associated macrophage (SAM-like) phenotype, analogous to macrophages that control fibrosis in the liver (45, 46), lung (47), heart, kidney, and skin (17) and this phenotype appears to be conserved across species and tissues and exhibits specificity to fibrotic disease (17, 47). The SAM-like population is characterized by expression of *Spp1, Gpnmb, Cd63* and *Trem2,* and *Mmps* and is predominantly monocyte-derived in other tissues with a role in extracellular matrix deposition and remodeling (46).

Of note, while we have identified that monocyte-derived macrophages give rise to lesion-resident macrophages with prodisease phenotypes, our previous studies using Ccr2^−/−^ mice demonstrated that loss of this recruitment axis resulted in development of more and larger lesions (15) as a result of a depleted monocyte-derived LpM pool. This also highlights distinct differences between lesion-resident and peritoneal cavity-resident macrophages in endometriosis. Akin to cancer, we have identified that cellular ontogeny accounts only for some of the transcriptional heterogeneity, thus signals received from the local environment are also major determinants of phenotype (48, 49).

Peritoneal cavity macrophages exhibit a highly specific transcriptional profile that is distinct from most lesion-resident macrophages. In our Endo-Intact dataset, a monocyte-derived LpM population was identified, congruent with the “protective” population of monocyte-derived LpM that we previously identified in our preclinical model and induced in endometriosis (15). Characteristic DEGs include *Pid1, Saa3, Apoe,* and *Lrp1*, each of which are associated with glucose and lipid metabolism and cholesterol efflux and may also play a role in fibrosis. Interestingly, studies have suggested that total cholesterol and triglycerides are up-regulated in the serum of women with endometriosis (50) and are positively correlated with disease severity (51). As cholesterol is a key precursor of steroid hormones, this points to a link between the increased estradiol biosynthesis in endometriosis lesions (52) and cholesterol metabolism in the peritoneal cavity. Thus, we postulate that the protective monocyte-derived LpM population reduces availability of triglyceride and cholesterol availability for lesion growth, as well as subsequently lowering local estrogen biosynthesis, thus reducing drivers for endometriosis maintenance and progression. Future work will focus on evaluating this hypothesis. In support of our findings, Jenkins et al reported that inflammatory macrophages abundant during mild zymosan-induced inflammation expressed genes that overlapped with LpM of recent monocyte origin in female mice, including Apoe (7).

In this study, we have used two variations of experimental endometriosis: Endo-Ovx (our original model) and Endo-Intact. We included the intact model following the realization that the transcriptomic profile of monocyte-derived LpM was masked in the Ovx model due to the large population on transitory LpM (monocyte-derived) present in both Sham and Endo-Ovx mice. These findings are consistent with previous studies demonstrating that surgery alters the immune composition and peritoneal macrophage dynamics in mice (6). Moreover, the Ovx model produces an artificial hormonal milieu, consisting of exogenous estrogen supplementation and the absence of progesterone. We suggest that future studies take this into consideration in their experimental design. We identified *Apoe* as a marker of monocyte-derived LpM in both variations of the endometriosis model. Quantification of Apoe+ LpM using flow cytometry revealed that this result was due to an influx of Apoe+ Tim4− LpM into the cavity in response to endometriosis, rather than overexpression of Apoe in this population. Of note, flow cytometry demonstrated that Tim4+ LpM actually express the highest levels of Apoe protein in the presence of endometriosis. Previous studies have demonstrated that endometriosis lesions promote differentiation of Tim4− monocyte-derived LpM into Tim4+ LpM (23); monocyte-derived Tim4− LpM elicited during inflammation resemble embryo-derived LpM but they are functionally distinct (7). This functional diversification includes a greater proliferative capacity, altered phagocytic properties, and enhanced cytokine secretion (e.g., TNF) (7). Thus, while our initial postulation that monocyte-derived LpM confer protective characteristics against development/persistence of endometriosis appears to remain true, our data suggest that it is the more differentiated phenotype (i.e., Tim4+) that enacts the protective characteristics.

In a gain-of-function experiment utilizing an Apoe mimetic peptide, we found that the size of endometriosis lesions was reduced. We also identified reduced collagen in Apoe treated lesions in support of previous studies indicating that Apoe can promote phagocytosis of type 1 collagen by macrophages in an LRP-dependent manner (24, 53). A lipid uptake assay revealed no significant differences between Tim4− and Tim4+ macrophages, thus further studies are required to comprehensively evaluate the underlying mechanisms of both populations of monocyte-derived LpM, these will include specific collagen uptake assays and assays that measure cholesterol efflux. Aside from its role in lipid metabolism, Apoe is also known to modulate both adaptive and innate immune responses in mice (54), and as the Apoe mimetic significantly elevated numbers of Tim4+ LpM, it may be suggested that Apoe has an autocrine role in regulating differentiation of Tim4− LpM into Tim+ LpM in the peritoneal cavity. Significant questions remain regarding its role in lipid metabolism and cholesterol efflux in the peritoneal cavity and the pathogenesis of endometriosis.

A recent scRNA-Seq study on human endometriosis-associated macrophages by Tan et al, identified heterogenous subpopulations of macrophages (26), and key macrophage populations identified in our mouse model are also present in the human endometriosis ecosystem. The concordance between human and mouse was remarkable, with a significant number of shared markers, indicating that we have a clinically tractable model for our study. For example, *FOLR2* and *MRC1* were top markers in their LYVE+ lesion-resident macrophages, indicating similarities with our TAM-like macrophages. *SPP1* was also a top marker in the activated macrophage population, and this population aligns with our SAM-like population. Similar to our data, Tan et al also identified infiltrated macrophages (with high expression of *CCR2*) and peritoneal macrophages (*ICAM2* and *FN1*). To further validate the concordance of mouse and human lesion-resident macrophages we performed integrative cross-species mapping (55). The analysis demonstrated coclustering of the major subpopulations for both human and mouse and highlighted proportions in each cluster. Overall, the similarity was beyond what we had predicted, but some deviations were apparent. For example, *SPP1+* (SAM-like) macrophages were expanded in the human, whereas *LYVE*+ (TAM-like) macrophages were expanded in mice. *VCAN*+ macrophages appeared to be almost uniquely human, and future work should focus on determining the function of these cells. In the peritoneal fluid, we have highlighted key similarities in macrophage populations between the human (25) and mouse, with the identification of equivalent populations to mouse LpM (prototypical tissue-resident population), transitory (differentiation intermediates), and monocyte-derived SpM. In the human, populations of *LYVE*+ and *VCAN+* peritoneal macrophages are present but significantly underrepresented in the mouse data, indicating these may play a more substantial role in human peritoneal physiology and future work should characterize function. Of note, it has not been possible to perform the cross-species mapping between the human and mouse on subjects with directly correlated hormonal status; specifically the human peritoneal fluid dataset was derived from a patient with endometriosis in the proliferative phase of their menstrual cycle and the patient had not been exposed to exogenous hormones for 6 mo (25). We mapped this dataset to both the Endo-Ovx (mice ovariectomized and supplemented with estradiol) and Endo-Intact (a pool of estrus stages) peritoneal fluid datasets and identified congruent populations with both. Key differences in the proportions of transitory pMs were evident, however we concluded that this was due to surgical rather than hormonal status. The human lesion data used in the study was derived from patients on a similar hormonal preparation (26) (most patients were on norethindrone/E2:1/20 contraceptive containing a progestin and an estrogen) and was mapped to lesions derived from the Endo-Ovx model (estrogen and no progesterone). Regardless of these differences, we identified macrophage phenotypes that clearly aligned between species suggesting that ectopic location and endometriotic niche shapes macrophage phenotype, with hormonal status playing a less prominent role.

In summary, we have demonstrated that macrophage subpopulations are transcriptionally diverse and that the major prodisease macrophage populations in endometriosis lesions are congruent with TAM and SAM and are largely monocyte-derived. We have identified that peritoneal macrophages are distinct from lesion-resident macrophages and exhibit both prototypical and protective phenotypes, with macrophage-derived Apoe as a key mediator of protection against development of endometriosis lesions. In the future, we propose that endometriosis researchers should look to cancer and other fibrotic diseases to exploit and repurpose macrophage-directed therapies that target these specific phenotypes and simultaneously modify the immune environment to promote accumulation of proresolving macrophages.

## Materials and Methods

### Mouse Model of Induced Endometriosis.

Endometriosis was induced in mice using a syngeneic model as previously described (12, 56).

### Flow Cytometry.

Lesions were dissected, pooled from each mouse, and placed in 2 mL ice-cold DMEM. Tissues were cut into small pieces using a scalpel and digested. RBCs were lysed and blocked. Cells were stained using a cocktail of antibodies detailed in *SI Appendix*, Table S1. Samples were processed using an LSRFortessa with FACSDiva software or FACSMelody with Chorus software (BD Biosciences) and analyzed with FlowJo v.9 software (FlowJo, Ashland, OR). For fluorescent activated cell sorting, red blood cell lysis, Fc blocking, and fluorescent staining were performed as previously described and samples sorted into pure cell populations based on cell surface marker expression using a FACS Aria Fusion (BD Biosciences).

### Single-Cell RNA-Sequencing and Analysis.

From donor endometrium (+4 to 6 h P4 withdrawal; n = 5 mice), endometriosis lesions (lesions from n = 10 mice), and peritoneal lavage from Sham (n = 5) and Endometriosis mice (n = 5), 200,000 CD45+ cells were FACS sorted into 1 mL PBS + 2% FBS using a FACS Fusion. Cells were barcoded using a 10X Genomics Chromium Controller^TM^ using established pipelines. Libraries were sequenced by Edinburgh Genomics using a NovaSeq 6000 sequencing system (Illumina®, San Diego, CA). Initial processing was performed using Cellranger (v2.1.1) mkfastq and count (aligned to mouse assembly mm10). For each dataset (filtered data from Cell Ranger 27 pipeline), potentially low-quality cells were filtered out using dataset-specific thresholds. Cell ranger metrics are available in *SI Appendix*, Table S2. Clustering and analysis of differential gene expression (DGE) was performed using Seurat (v4.4.0 to v5.0.0) in R (v4.3.2). Further details are provided in *SI Appendix*, *Methods*, including methods for KEGG, GO, and cross-species mapping.

### Cell Culture and In Vitro Functional Assays.

Macrophage CM were generated from Folr2+ and Folr2− macrophages and used to assess impact of macrophage-derived secreted factors on the formation of angiogenic networks (HUVECs), and induction of fibrosis-associated genes in huESCs. Further details are available in *SI Appendix*, *Methods*. We also evaluated the function of Tim4+ and Tim4− LpM using a phagocytosis assay (Cayman Chemical) and lipid uptake assay (*SI Appendix*, *Methods*).

### RT-qPCR.

RNA was extracted from FACS sorted macrophages using RLT(lysis) buffer and an RNAeasy Kit (Qiagen, Hilden, Germany), RNA was amplified or cDNA generated according to the manufacturer’s instructions and qPCR performed using Express qPCR mastermix (further details in *SI Appendix*, *Methods*).

### Immunofluorescence.

Immunofluorescence was carried out as previously described (14, 15, 57). To identify TAM- and SAM-like macrophages in mouse lesions, sections were stained for F4/80 (macrophages) in combination with Gas6 (TAM) or Spp1 (SAM). In human lesions, macrophages were identified using CD68 in combination with Gas6 or Spp1 (*SI Appendix*, *Methods*).

### Masson-Trichrome Stain.

Paraffin-embedded mouse lesion and endometrium tissue sections (3 µm) were stained using the Abcam Trichrome kit (ab150686 Trichrome stain) following the protocol provided.

### Fiji Analysis.

Masson-trichrome images were obtained using the Leica DMi8 with an 10X objective. Collagen intensity analysis was ascertained in Fiji image analysis software (Image J v1.53) by isolating stained area of blue coloration (collagen) using color thresholding, creating a mask of this selection, and utilizing particle analysis to measure the total area of the mask. This was then presented as a proportion of the total area of the lesion.

#### Statistical Analysis.

Statistical analysis was carried out in GraphPad Prism 10.0. Data were analyzed for normality using a Shapiro–Wilk normality test. If data were normally distributed, either an ANOVA with a Tukey’s post hoc test (more than two samples) or a *t* test (two samples) was performed. If data were not normally distributed, nonparametric tests were used, either Kruskal–Wallis with a Dunn’s post hoc test (more than two samples) or a Mann–Whitney U test (two samples). Statistical significance was reported at *P* < 0.05.

## Supplementary Material

Appendix 01 (PDF)

Dataset S01 (XLSX)

Dataset S02 (XLSX)

Dataset S03 (XLSX)

Dataset S04 (XLSX)

Dataset S05 (XLSX)

Dataset S06 (XLSX)

Dataset S07 (XLSX)

Dataset S08 (XLSX)

Dataset S09 (XLSX)

Dataset S10 (XLSX)

## Data Availability

The sequencing data are deposited in the GEO repository (https://www.ncbi.nlm.nih.gov/geo/) under accession number (GSE274438) (58). Code is available on GitHub (59). All other data are included in the manuscript and/or supporting information.
