# Supplementary material for: Single-cell analysis identifies distinct macrophage phenotypes associated with prodisease and proresolving functions in the endometriotic niche
Source: Proc Natl Acad Sci U S A. 2024 Sep 10;121(38):e2405474121. doi: 10.1073/pnas.2405474121 (PMC11420174; doi:10.1073/pnas.2405474121)
Supplement: Supplementary file 1 — Appendix 01 (PDF) [file pnas.2405474121.sapp.pdf]

SI appendix

Methods

Animals and reagents. Wild-type C57BL/6JOlaHsd and FVB female mice were purchased from Harlan (Harlan Sprague Dawley Inc, Bicester, UK) at 8-12 weeks of age. B6.Cg-Tg(Csf1r-EGFP)1Hume/J (MacGreen) express enhanced green fluorescent protein (EGFP) under control of the Csf-1r promoter[1] and FVB-Tg(CAG-luc,-GFP)L2G85Chco/J were bred in house[2]. Mice were maintained at the University of Edinburgh or the University of Warwick. All animal work was licensed and carried out in accordance with the UK Home Office Animal Experimentation (Scientific Procedures) Act 1986 and the work licensed under PPL 70/8731 and PP0568394 (E.G). Mice had access to food and water ad libitum and were kept at an ambient temperature and humidity of 21°C and 50% respectively. Light was provided 12 hours a day from 7am-7pm. To visualize bioluminescent endometriosis lesions, the substrate D-luciferin (1.5 mg/100 µl in PBS; Sigma-Aldrich, Dorset, UK) was injected s.c. prior to imaging under anaesthesia using a PhotonIMAGER (Biospace Lab, Paris, France)[2]. For Apoe experiments, mice were injected intra-peritoneally with COG133 (Generon A1131; 3µM in 200µl sterile H20) or vehicle (sterile H20) daily from onset of endometriosis to 14 days post induction.

Mouse model of induced endometriosis. The model aims to mirror the process of 'retrograde menstruation'. In brief, donor mice were induced to undergo a 'menses'-like event by removing the ovaries and exposing the mice to a hormonal schedule similar to a truncated menstrual cycle and a stimulus that causes the endometrial stromal cells to undergo decidualization[3]. Following P4 withdrawal the endometrial lining begins to shed. 4-6hrs after withdrawal of P4, the 'menses'-like endometrium was collected and injected into ovariectomized mice supplemented with oestradiol valerate[3]. Lesions were recovered that contain stoma +/- epithelial cells and immune cell influx[4]. For some experiments endometrial tissue or FACS sorted LpM were isolated from MacGreen mice and transferred to wild-type recipients during induction of endometriosis to allow the isolation of endometrial-derived or peritoneal-derived macrophages to be isolated from resultant lesions as previously described[5]. For some experiments recipient mice were left with their ovaries intact and did not receive exogenous oestradiol valerate. For non-invasive bioluminescent imaging of lesions, endometrial tissue from CAG-luc-eGFP mice was injected into the peritoneal cavity of wild-type FVB mice. Lesions were collected 2 weeks following tissue injection into ice-cold DMEM. Peritoneal lavage was recovered by injecting 7 ml ice-cold DMEM into the peritoneal cavity followed by gentle massage and recovery.

Flow cytometry. Lesions were digested with 1 unit of Liberase DL, 1 unit of Liberase TL (Roche) and 0.6 mg DNAse enzymes. The tissue and enzymes were incubated for 45 minutes at 37°C, with vortexing every 5 minutes. Following digestion, samples were filtered through 100µM filters. Red blood cells were lysed from peritoneal lavages and cells derived from the endometrium or lesions, and approx. 10^6 cells per sample were blocked with 0.025mg anti-CD16/32 (clone 93; BioLegend, San Diego, CA, USA) and then stained. Brilliant™ violet stain buffer (BD Biosciences) was included when required. For intracellular staining, cells were fixed in 4% PFA after staining for cell surface markers, permeabilised (0.2% Tween-20 in PBS) and stained using an Apoe-PE antibody). Fluorescence minus one (FMO) and unstained controls were used to validate gating strategies. Just prior to analysis on the flow cytometer, DAPI and 123count eBeads (Thermo Fisher Scientific) were added to samples. Following processing , analysis was performed on single, live cells determined using scatter height vs. area and negativity for live or dead (DAPI or alternative viability dye).

Bioinformatics analysis. In Seurat, we preferentially, we selected cells where at least 200 genes were detected and only genes that were expressed in at least 3 cells were included in down-stream analysis. To reduce the risk of including low-quality cells we integrated our datasets and performed quality control to filter out cells with high mitochondrial gene expression (8%), followed by data normalisation and scaling as per standard Seurat workflow (Stuart and Satija, 2019). As the samples in each dataset were all processed on the same day, cartridge and sequenced on the same lane, batch correction was not utilised. To visualize the population dynamics and reduce dimensionality of our merged dataset, we ran a principal component analysis (PCA) on the normalized gene matrix of the top 4000 most variable genes. Cell clustering was visualized using Uniform Manifold Approximation and Projection (UMAP; PCAs 11, dims 11, resolution 0.6 (aggregate, Fig.1), PCAs; 17, dims; 17, resolution 0.6 (PF; Fig.3)). The function Clustree[6] was used to visualise the relationships between clusters and how these changed at different resolutions. This also allowed for the identification of distinct and unstable clusters. The Seurat function 'FindAllMarkers' was used to identify marker genes for each cluster in the UMAP projection according to the inbuilt Wilcox statistical test. To identify potential doublets from our dataset, we used the 'doublet finder' function to exclude these cells and repeated normalisation, feature selection, scaling and UMAP projection and cluster marker identification as described above. Ultimately, we retained 1295 cells from donor endometrium, 5560 cells from lesions, 4966 cells from sham peritoneal fluid and 6265 from endometriosis peritoneal fluid (Endo-Ovx). For the intact dataset we retained 3895 (Naïve PF) and 2583 (Endo-Intact). We used a manual approach to label cell clusters using canonical markers derived from comprehensive literature review and specialized databases

housing cell type marker genes including PanglaoDB and CellMarker 2.0. These were used alongside Seurat's 'FindMarkers' function, to assign cell type identities to clusters in the UMAP projection.

KEGG and GO. The statistical analysis and visualisation of functional profiles for each gene cluster was analysed using the function compareCluster from the package *clusterProfiler*. First, data sets were subset to include only the macrophage subpopulations. Differentially expressed genes (DEGs) were then generated using Seurat's FindAllMarkers function and subset for markers that were upregulated with adjusted p values lower than 0.05. This list was used for the geneCluster argument of compareCluster. For the identification of specific Gene Ontology (GO) and Kyoto Encyclopedia of Genes (KEGG) terms, a custom universe was also created which included only genes that were expressed in the subset data and this list was used in the universe argument of compareCluster. For GO enrichment analysis and KEGG enrichment analysis, the fun argument was set to enrichGO or enrichKEGG respectively.

Cross-species analysis. Analyses were performed on mouse lesion and peritoneal lavage data from the Endo-Ovx dataset above, and human data retrieved from the publicly available datasets of Tan et al (peritoneal endometriotic lesions)[7] and Zou et al. (endometriosis peritoneal fluid)[8] using the guideline best practices for single-cell analysis described by Heumos et al[9]. Raw scRNA-seq data was demultiplexed, aligned to reference genomes (h38, mm10) and processed using Cell Ranger Software as above. Analysis of gene-barcode matrices were performed in Seurat (5.0.0) in R (4.3.2) with Seurat objects (v5) created using cells with > 200 genes, and genes expressed in > 3 cells. Quality filtering of scRNA-seq data was performed at the per sample level to remove cells of low quality using multiple filtering parameters of mitochondrial percentage (< 20), number of genes detected (> 350), gene expression counts (> 1000 in human samples; > 350 in mouse samples due to reduced sequencing depth), and log10GenesPerUMI (> 0.8). The packages scDblFinder, SoupX, and cc.genes (Seurat) were used to identify and remove doublets, estimate ambient RNA contamination, and perform cell cycling scoring, respectively[10, 11].

Parallel analysis. Seurat package was used to normalise individual expression matrices using the 'NormalizeData' and 'ScaleData' functions, with the 'FindVariableFeatures' function implemented to select the top 2000 variable genes for PCA analysis within the 'RunPCA' function. The 9 human (peritoneal) lesion Seurat objects were integrated to batch correct for inherent patient differences, the other 3 datasets are composed of individual Seurat objects. The Seurat functions 'RunUMAP', 'FindNeighbours' and 'FindClusters' were used for visualisation and clustering. The parameters for

clustering lesions were as follows: mouse; 30 PCAs, 1:30 dims, resolution 0.5, human; 30 PCAs, 1:30 dims, resolution 0.65; combined; 50 PCAs, 1:30 dims, resolution 0.4. The parameters for PF were as follows: mouse; 30 PCAs, 1:30 dims, resolution 0.4, human; 30 PCAs, 1:30 dims, resolution 0.7, combined; 50 PCAs, 1:30 dims, resolution 0.3. As the mouse peritoneal fluid and lesion datasets contained only CD45+ cells, human datasets were subset on CD45+ expressing clusters, followed by re-running of visualisation and clustering steps. Seurat 'FindAllMarkers' Wilcoxon rank-sum tests implemented in a one-versus-all fashion were used to identify marker genes for manual annotation according to canonical biomarkers identified in literature study. DEG analysis was performed using the MAST functionality in Seurat 'FindAllMarkers' to account for within-sample correlation. Hypergeometric tests to compute the significance of overlapping DEGs between mouse and human datasets were performed in R (4.3.2) on homologous DEGs with a > 1.5 or < -1.5 log2 fold change, against the hypergeometric distribution conferred by random iterative sampling of the overlap occurring when sampling the same number of DEGs from each species-specific homologous genomic background in 100000 simulations to account for the different number of genes original R objects.

Integrative cross-species mapping. Cross species integration of single-cell RNA-sequencing data was performed in Seurat 5.0.0 using the Seurat V4 CCA O2O strategy described by Song et al. due to the high integrated scoring of species mixing and biological conservation[12]. Homology mapping was performed using one-to-one orthologous genes between human and mouse samples. Prior to creation of Seurat objects, human genes within the gene-barcode matrices were translated to their mouse one-to-one orthologs using the convert_human_to_mouse_symbols function in the nichenetr R package[13]. Human genes without mouse one-to-one orthologs were removed, and all datasets were then subset on genes common to both human and mouse datasets. Seurat objects were then created, processed, and visualised as prior, with integration of human and mouse macrophage clusters performed using Seurat v4 anchor based CCA integration, followed by marker gene and DEG analyses as described above. For validation of integrated clustering, the Seurat 'WhichCells' functionality was used to identify cell barcodes of specific clusters in the single-species analyses, with these cells then projected onto UMAPs in the cross-species integrated analyses.

Human samples

Endometriotic lesion biopsies (peritoneal and endometriomas) were collected from patients enrolled in the EndoWar study (University of Warwick REC: 19/LO/1647) who provided informed consent and were undergoing laparoscopy for suspected endometriosis. Tissues and clinical metadata were de-identified and collected in accordance with the WERF EPHect guidelines[14, 15]. For the human samples

included in the immunofluorescence study (n=10), women were aged between 25 and 41 (mean age 30.8). We stained n=6 superficial peritoneal lesions and n=4 ovarian endometriomas. N=5 women were on hormonal medication, n=5 were not on hormones. Of those not on hormones n=2 were secretory phase samples and n=3 were proliferative phase samples.

For the isolation of human endometrial stromal cells (huESCs), endometrial biopsies (n=2) were received from Jan Brosens (University of Warwick; REC 18/WA/0356). Biopsies were collected for research purposes with written informed consent obtained from patients prior to tissue collection in accordance with the guidelines of the Declaration of Helsinki, 2000. Endometrial biopsies were obtained using a Endosampler (Medgyn, IL, U.S.A.), during the secretory phase of the cycle from patients attending a dedicated research implantation clinic (and were undergoing investigation of recurrent pregnancy loss). All samples were collected at the University Hospitals Coventry and Warwickshire (UHCW) National Health Service (NHS) Trust, Coventry, UK.

Cell culture

Generation of conditioned media from FACS sorted macrophages. Folr2+ and Folr2– macrophages were isolated by fluorescent-associated cell sorting (FACS) from peritoneal fluid and lesions from mice with experimental endometriosis (n=7) using a BD FACS Fusion Aria cell sorter. Sorted macrophages were seeded at a density of 10,000 cells per 50 µL of recovery media (DMEM-hi-glucose, 2% HI-FBS, 25 mM HEPES, 50 µM β-mercaptoethanol, 1mM Sodium Pyruvate, 1x antibiotic-antimycotic, 1x non-essential amino acids), and cultured for 24 hrs at 37°C (5% CO_2) humid incubation. Following overnight macrophage cell recovery culture, the media was aspirated, and the macrophages were replenished with fresh low-serum media (DMEM-hi-glucose, 0.2% HI-FBS, 25 mM HEPES, 50 µM β-mercaptoethanol, 1mM Sodium Pyruvate, 1x antibiotic-antimycotic, 1x non-essential amino acids) to create conditioned medium. Macrophage conditioned media was then harvested after 24 hrs overnight incubated culture at 37°C (5% CO_2) humidity and cryopreserved at –80°C for later use.

HUVEC cell culture. Human umbilical vein endothelial cells or HUVECs were derived from a pool of donors (Promocell, #C-12203) and expanded to passage 5 in VEGF-rich complete media (Promocell, #C-22011) at 37°C, 5% CO_2, humidified incubation.

Endometrial stromal cell culture. Human endometrial stromal cells (huESCs) were derived from biopsies as previously described[81]; in brief, different endometrial cellular components were dissociated by mincing the tissue with a scalpel blade for 5 minutes then digested by shaking

incubation in an enzymatic cocktail consisting of 5mL phenol red-free Dulbecco's Modified Eagle Medium (DMEM)/F12, 0.5 mg/mL collagenase I and 0.1 mg/mL DNAse I (Sigma) at 37°C, 5% CO2, humidity for 1 hour. Human ESCs were then specifically separated from the resulting homogenate by washing in complete growth medium (DMEM/F12 containing 10% dextran-coated charcoal stripped FBS (DCC-FBS), 1% penicillin-streptomycin, 2 mM L-glutamine, 1 nM E2 (Sigma-Aldrich) and 2 mg/ml insulin (Sigma-Aldrich) followed by filtration through a 40 µm cell strainer, where huESCs were obtained in the flowthrough. The flowthrough was further washed in complete growth medium and centrifuged (400xg 5 minutes, RT). Resulting cell pellets were resuspended in 10 mL complete growth medium and seeded in tissue culture flasks. Any contaminating (non-adherent) cell were removed by replacing the medium following overnight cell culture. The cell culture medium was subsequently replenished at 48-hour intervals and sub-confluent monolayers of huESC were passaged at a 1:3 ratio using 0.25% Trypsin-EDTA.

Ex vivo functional assays

Phagocytosis assay. Mouse peritoneal lavage was collected and prepared for FACS with blocking and staining steps as previously described. Samples were stained with a panel of antibodies as show in SI Table 9. The cell suspension (100µl) at a concentration of 1-5x10^6cells/ml was incubated with a 1:100 final dilution of latex beads-rabbit IgG-FITC complex from the Cayman Chemical Phagocytosis Assay Kit (IgG FITC) for 1 hour at 37°C in FACS tubes. To assess phagocytic activity, the suspensions were pelleted at 400 x g for 5 minutes and resuspended in 300µl of flow buffer (PBS+2%BSA) before being analysed on the LSR Fortessa™ with FACS Diva software and analysed with FlowJo as previously described. The gating strategy live, single, CD45+, lineage-, Cd11b+, $MHCII^{lo}$, F4/80^{hi} was used to identify the large peritoneal macrophages (LpM). Monocyte-derived LpM were $Timd4^{lo}$ and embryo-derived LpM were $Timd4^{hi}$ and sorted using this distinction. The internalisation of the latex beads was quantified as a measure of macrophage phagocytosis by noting the fluorescence intensity of FITC and compared between monocyte and embryo-derived (long-lived) macrophages.

Lipid uptake assay. FACS sorted Tim4- and Tim4+ LpM were recovered in overnight culture at 37°C incubation (2% FBS-HI, DMEM, HEPES, antibiotic-antimycotic, 1x non-essential amino acids, 25 mM sodium pyruvate, 50 uM B-mercaptoethanol). The following day, LpM were co-cultured in low-serum media (0.2% FBS-HI, DMEM, HEPES, 0.1x antibiotic-antimycotic, 0.1x non-essential amino acids, 2.5 mM sodium pyruvate, 5 uM B-mercaptoethanol) with Ox-LDL (oxidised low-density lipoprotein conjugated to DiL (Thermo Fisher Scientific) for 5 hours, as determined by a previous timecourse. Excess OxLDL-DiL was removed with PBS washes and, cells were fixed in 4% PFA for 10

minutes, washed in PBS and counterstained using DAPI for nuclei visualisation. Covered in PBS, cells were then imaged on an EVOS M7000 microscope. DAPI positive and CTFR dye positive cells identified LpM. The number of DiL positive LpM were represented as a percentage of total LpM.

Endometrial stromal cell gene expression. A collation of 20,000 huESCs derived from two participants were seeded in complete growth medium into a 96-well plate and allowed to expand for 48 hours at 37°C, 5% CO2, humidified incubation. This was followed by overnight serum starvation in low serum growth medium (DMEM/F12 containing 2% dextran-coated charcoal stripped FBS (DCC-FBS), 1% penicillin-streptomycin, 2 mM L-glutamine, 1 nM E2 (Sigma-Aldrich) and 2 mg/ml insulin (Sigma-Aldrich)). Following overnight serum-starvation, huESCs were cultured in the presence of macrophage conditioned medium (CM) derived from macrophages isolated from mice with endometriosis and naïve controls over a predetermined period of 3 days. Macrophage CM was pre-diluted at a ratio of 1:4 in low-serum huESC culture medium. Untreated and TGF-β1 (5 ng/mL, 10 ng/mL)-treated cells were used as negative and positive controls, respectively, and were cultured in stock CM culture medium diluted 1:4 in low-serum huESC culture medium. HuESCs were cultured in macrophage conditioned media for 3 days at 37°C, 5% CO2, humidified incubation before harvesting.

Angiogenesis assay. HUVECs underwent VEGF-starvation for 24 hrs prior to use in an angiogenesis assay (Promocell, #C-22010). A fluorescent Calcein stain in Ca2+ and MG2+ free HBSS (40 minutes, 37°C, 5% CO2 humidified incubation) was applied to HUVECs before seeding 15,000 cells resuspended in low-serum media (0.5% FBS and VEGF-negative, Promocell #C-22010 diluted 1:3 in basal media # C-22010B) on to a layer of Matrigel BME (25 µL, Corning, #356231) in a 96-well transwell culture arrangement. Conditioned media (200 µL) was diluted 1:9 in low-serum (0.5% FBS and VEGF-negative) media, and added to the lower chamber. The formation of vascular networks were imaged at timepoints 2, 4, 6, 8, 12 and 16 hrs on an EVOS M7000 Imaging system. Phase contrast images were then analysed in Fiji using the pre-validated Angiogenesis Analyzer workflow.

Real-time qPCR. Concentration and purity were assessed using a Nanodrop 1000 (Thermo Fisher Scientific). For FACS sorted cells RNA was amplified cDNA synthesized using a SeqPlex RNA Amplification kit (Merck Life Science, UK) as per manufacturer's instructions. For endometrial stromal cells, cDNA was synthesized using SuperScript Vilo Enzyme (Thermo Fisher Scientific) with 100 ng starting template in a 20µl reaction. A standard curve was generated by pooling samples and performing four 10-fold dilutions. PCRs (10µl) were performed using validated Taqman™ qPCR assays (20mM; Applied Biosystems, UK) and Express qPCR Supermix (Thermo Fisher Scientific). cDNA was

added at 1μl per reaction and thermal cycling conditions were performed on a 7900 Fast real-time PCR machine (ThermoFisher Scientific) in 384-well plates with technical duplicates performed. 18S (Thermo Fisher Scientific) was selected as the reference gene. Data were analysed using the relative standard curve method, and samples were normalized to 1 consistent sample.

Immunofluorescence. Immunofluorescence was carried out as previously described[5, 16, 17]. In brief, 5 µm thick sections of FFPE lesions collected from the mouse Endo-Ovx endometriosis model were stained by dual immunofluorescence to identify F4/80+GAS6+ TAM, and F4/80+SPP1+ SAM. Sections were adhered to microscopic slides by overnight incubation at 65°C. After dewaxing and rehydrating in gradient solutions of ethanol, sections underwent antigen retrieval in trypsin (Sigma, #, 1g/ml, 10 minutes, 37°C). Sections were permeabilised in 0.25% triton for 30 minutes at room temperature (RT). Endogenous peroxidases were blocked in 3% hydrogen peroxidase (30 minutes, RT), and non-specific binding was completed using normal goat serum (NGS) (30 minutes, RT). The first primary antibody (rat anti-F4/80 (eBioscience, #14-4801-82, dilution 1:100)) was applied overnight, followed by F4/80 antigen detection using a goat polyclonal anti-rat antibody conjugated to horseradish peroxidase (IMPRESS, # MP-7404), followed by a 10-minute incubation in Tyramide red (1:50 in diluent). Sections underwent a repeat antigen retrieval that was optimised for the second primary antibody in the dual immunofluorescence strategy. Gas6 antigens were retrieved in citrate buffer (pH6). Spp1 antigens were retrieved in Tris-EDTA buffer (pH8). As before, sections were blocked for endogenous peroxidases and non-specific binding before applying primary antibodies rabbit polyclonal anti-Gas6 (ThermoFisher, # PA5-79300, dilution 1:100) and rabbit polyclonal anti-Spp1 (ThermoFisher, # PA5-34579, dilution 1:100). Gas6 and Spp1 antigen detection was achieved using a goat polyclonal anti-rabbit antibody conjugated to horseradish peroxidase (IMPRESS, # MP-7451), followed by a 10-minute incubation in Tyramide green (1:50 in diluent). Washes were completed in PBS-Tween in between steps. Sections were finally mounted in an anti-fading mounting medium with DAPI (Vectashield, # H-2000). Antibodies were validated using single stain controls, and the omission-of-one primary antibody controls in a dual stain. Images of whole lesions were captured using a 20X objective on an EVOS M7000 (Invitrogen, UK) imaging system.

Dual immunofluorescence to identify CD68+GAS6+ TAM and CD68+SPP1+ SAM in human lesions was performed as follows: Sections (3µm) were deparaffinised and antigen retrieval performed in TRIS-EDTA buffer pH9. After endogenous peroxide blocking (using 3% hydrogen peroxide) and species-specific blocking (using normal goat serum 5% in 2% bovine serum albumin), sections were stained with monoclonal mouse anti-human CD68 clone PG-M1 - 1:100 (Dako Omnis: GA61361-2). Second

antigen retrieval and blocking were performed as described above and subsequent staining was performed with either rabbit anti-Gas6 - 1:100 (PA5-79300) or rabbit anti-SPP1 - 1:100 (PA5-34579). Secondary antibody and fluorescent staining was performed using the ImmPRESS IgG polymer detection kits (MP-7451, MP-7452) and Tyramide TSA plus kit with either Fluoresciene or Cy5. Autofluorescence quenching was performed with the TrueBlack® Lipofuscin Autofluorescence Quencher (Biotium: 203007) according to manufacturer's instructions. Slides were mounted with VECTASHIELD PLUS Antifade Mounting Medium with DAPI (Vector Laboratories: H-1000-10) and imaged on an EVOS M7000 (Invitrogen, UK) microscope.

SI Figures and legends

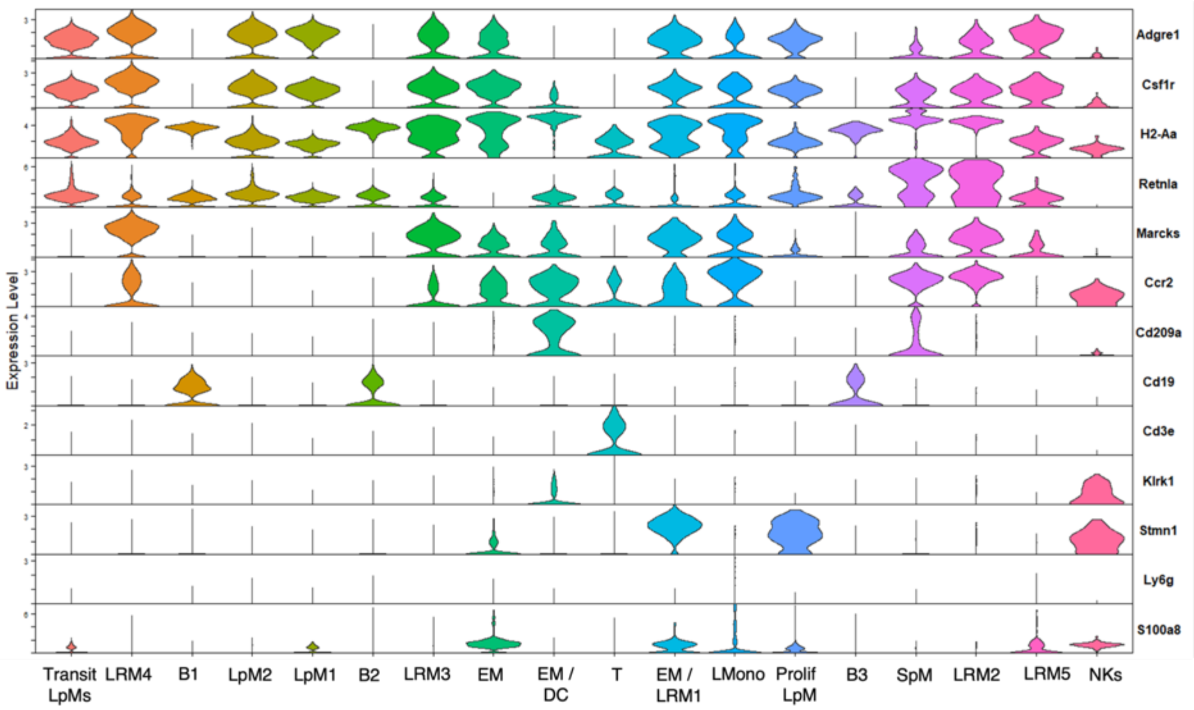

Figure S1: Canonical markers used for assigning cell identity. Violin plots showing expression of canonical markers used to identify cell clusters in Figure 1.

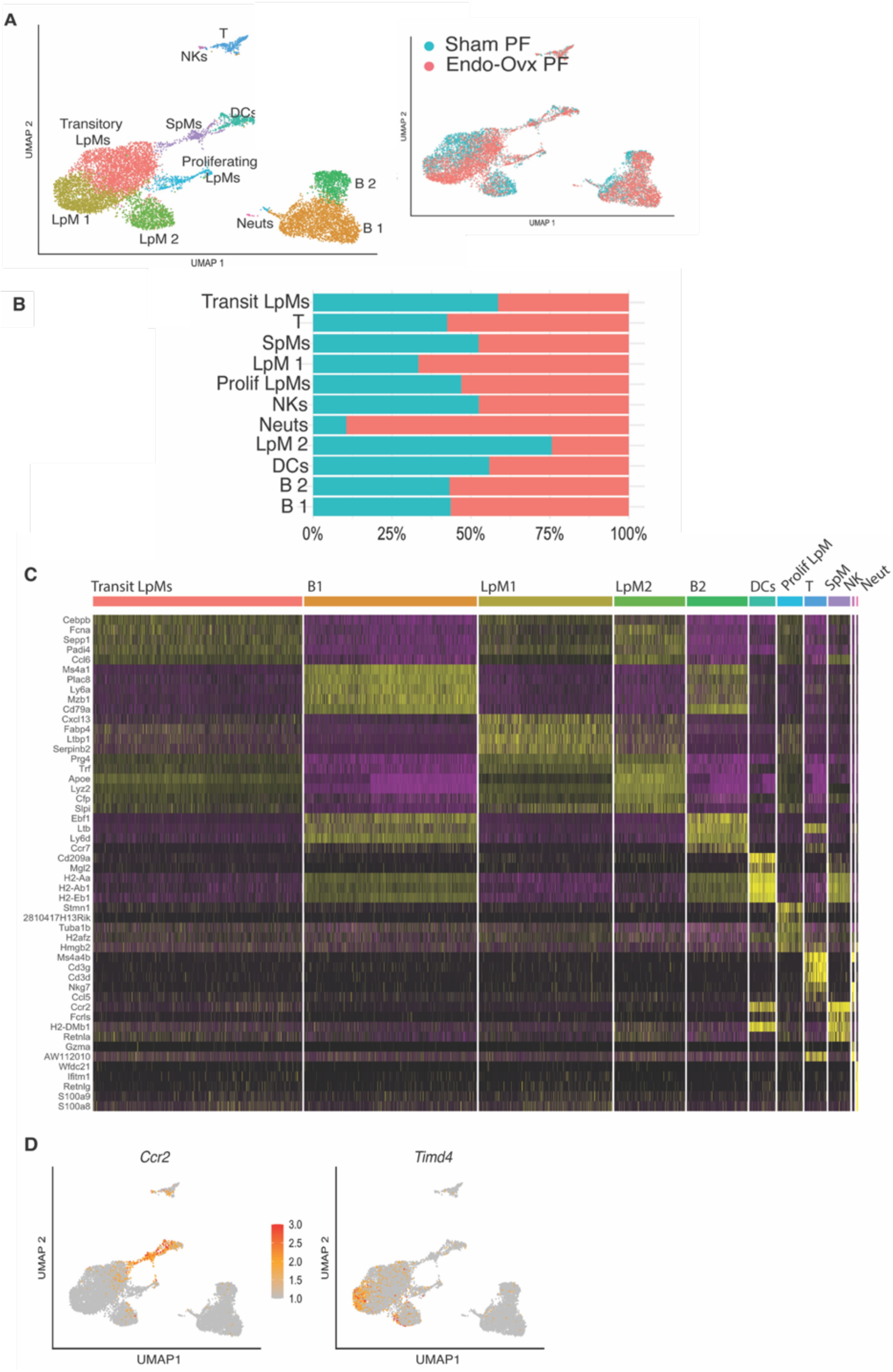

Figure S2: Transcriptomic heterogeneity of peritoneal macrophages in an ovariectomised model of induced endometriosis. A) UMAP projection of CD45+ cells isolated from peritoneal lavage recovered from Sham and Endo-Ovx mice. Inset shows UMAP based on library ID. B) Bar chart showing cluster membership of different sample types. C) Heatmap showing top 5 DEGs for each cluster. D) Feature plot of *Ccr2* and *Timd4*.

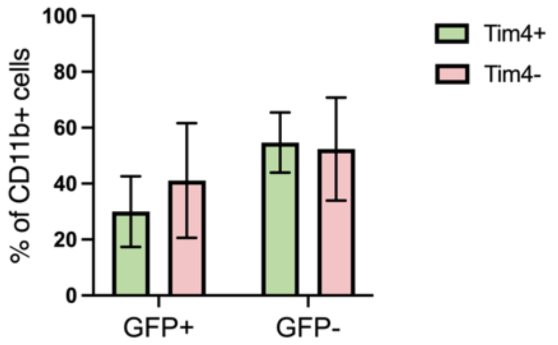

Figure S3: Analysis of Tim4+ and Tim4- LpM incorporation into endometriosis lesions. Tim4+ or Tim4- LpM recovered from MacGreen mice were adoptively transferred into wild-type mice inoculated with wild-type endometrium and GFP+ and GFP- cells recovered from lesions after two weeks. Graph shows quantification of cells recovered (Tim4+ n=7 mice, Tim4- n=3 mice).

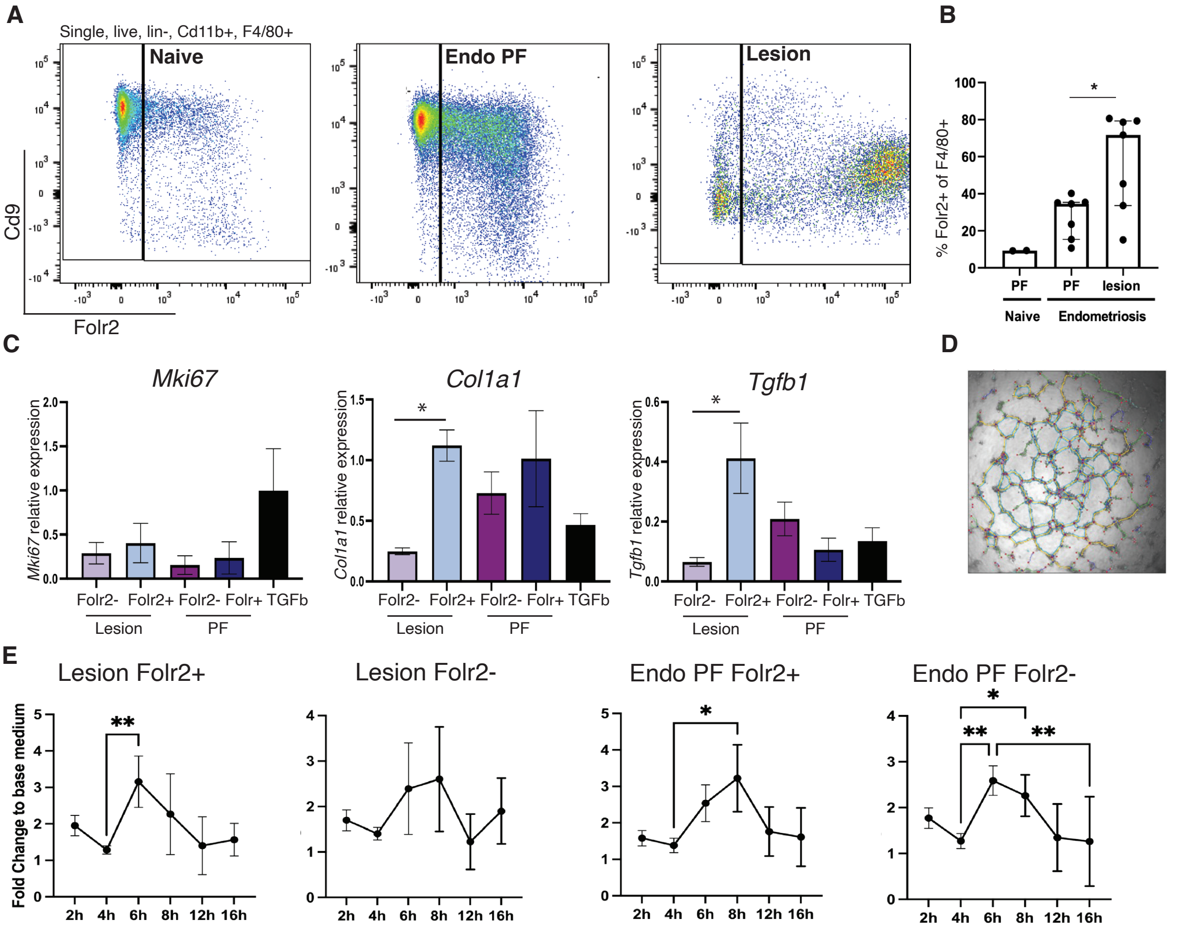

Figure S4: Lesion-resident Folr2+ macrophages exhibit 'pro-disease' properties. A) Representative flow plots showing Folr2 expression on F4/80+ macrophages isolated from PF from naïve mice, and PF and lesions recovered from endometriosis mice (n=7 mice). B) Quantification of flow data, showing numbers of Folr2+ macrophages in PF and lesions. FACs sorted Folr+ and Folr2- macrophages derived from PF and lesions of mice with endometriosis were cultured and conditioned media (CM) collected (24h). Data presented are median with interquartile range. C) To assess the impact of endometriosis-associated Folr2+ macrophages on proliferation and ECM deposition / fibrosis, primary human (eutopic) endometrial stromal cells (ESCs; from women without endometriosis) were exposed to macrophage CM (24h; diluted 1:1 in minimal media) and the mRNA concentration of *Mki67*, *Col1a1* and *Tgfb1* assessed by QPCR. Data presented are mean ± SEM. Human umbilical vein endothelial cells (HUVECs) plated onto Matrigel were also exposed to macrophage CM and network formation assessed. D) Representative image of endothelial cell networks (meshes, branches, junctions, nodes and segments were quantified). E) Quantification of meshes suggests that lesion-resident Folr2+ induce a rapid increase in angiogenesis compared to lesion-resident Folr2-, whereas PF Folr2- macrophages have a more potent effect on angiogenesis compared to PF Folr2+. Data shown are mean

± SD. Statistical analysis was performed using a Kruskall-Wallis and a Dunn's multiple comparison test. In (E) comparisons were carried out using a Friedman test combined with a Dunn's multiple comparison test. *: $p<0.05$, **: $p<0.01$.

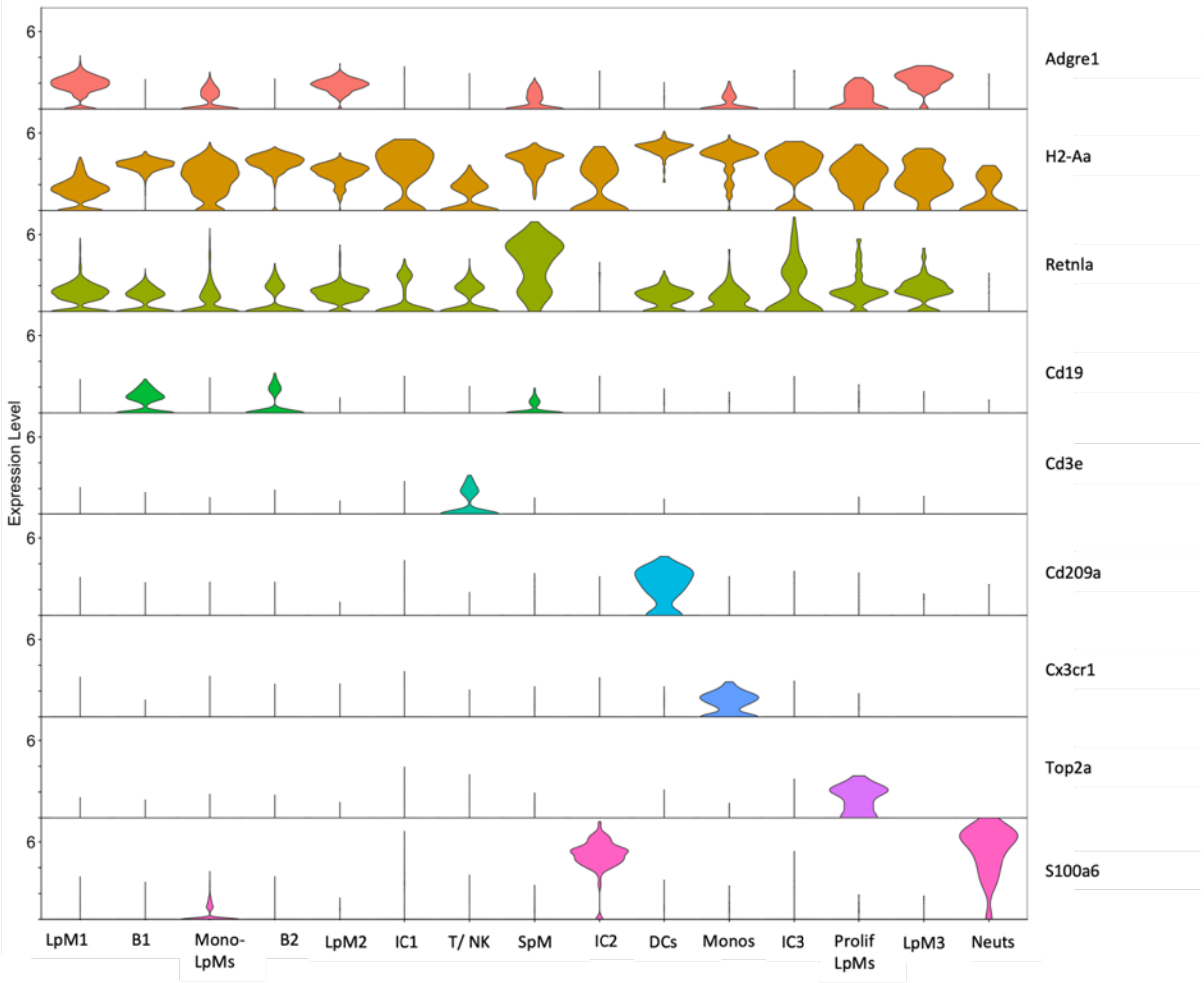

Figure S5: Canonical markers used for assigning cell identity. Violin plots showing expression of canonical markers used to identify cell clusters in Figure 3.

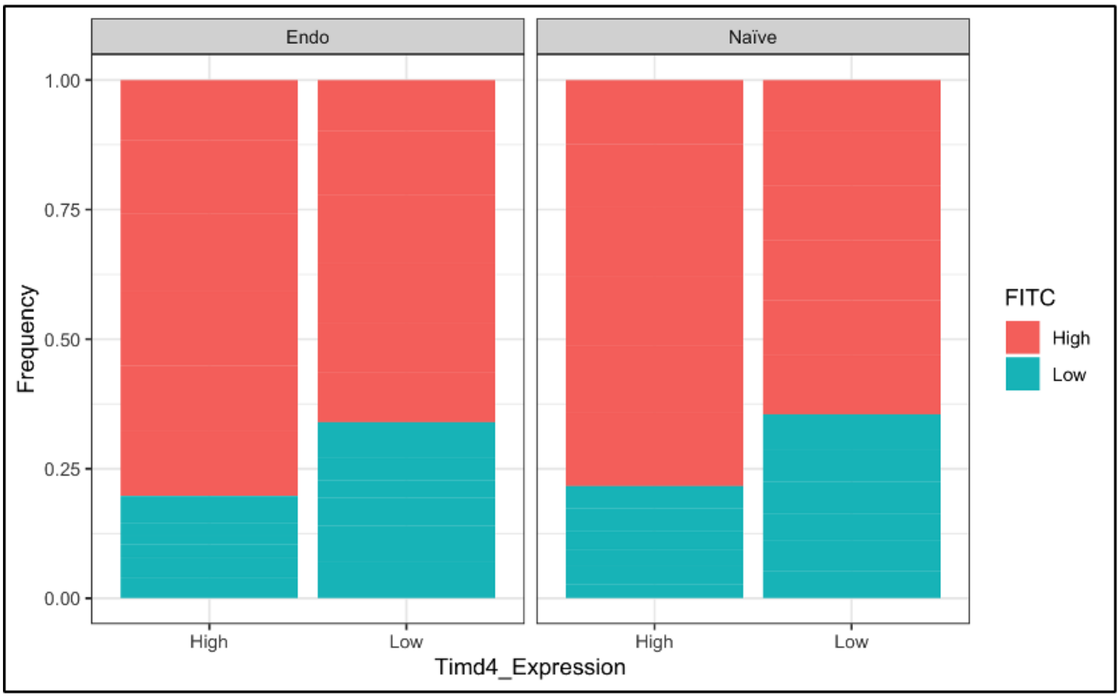

Figure S6: Analysis of phagocytic activity in Tim4^{hi} and Tim4^{lo} macrophages isolated from the peritoneal cavity of mice with and without induced endometriosis. Tim4^{hi} and Tim4^{lo} peritoneal macrophages were FACs sorted and incubated with FITC beads and phagocytic uptake measures using flow cytometry.

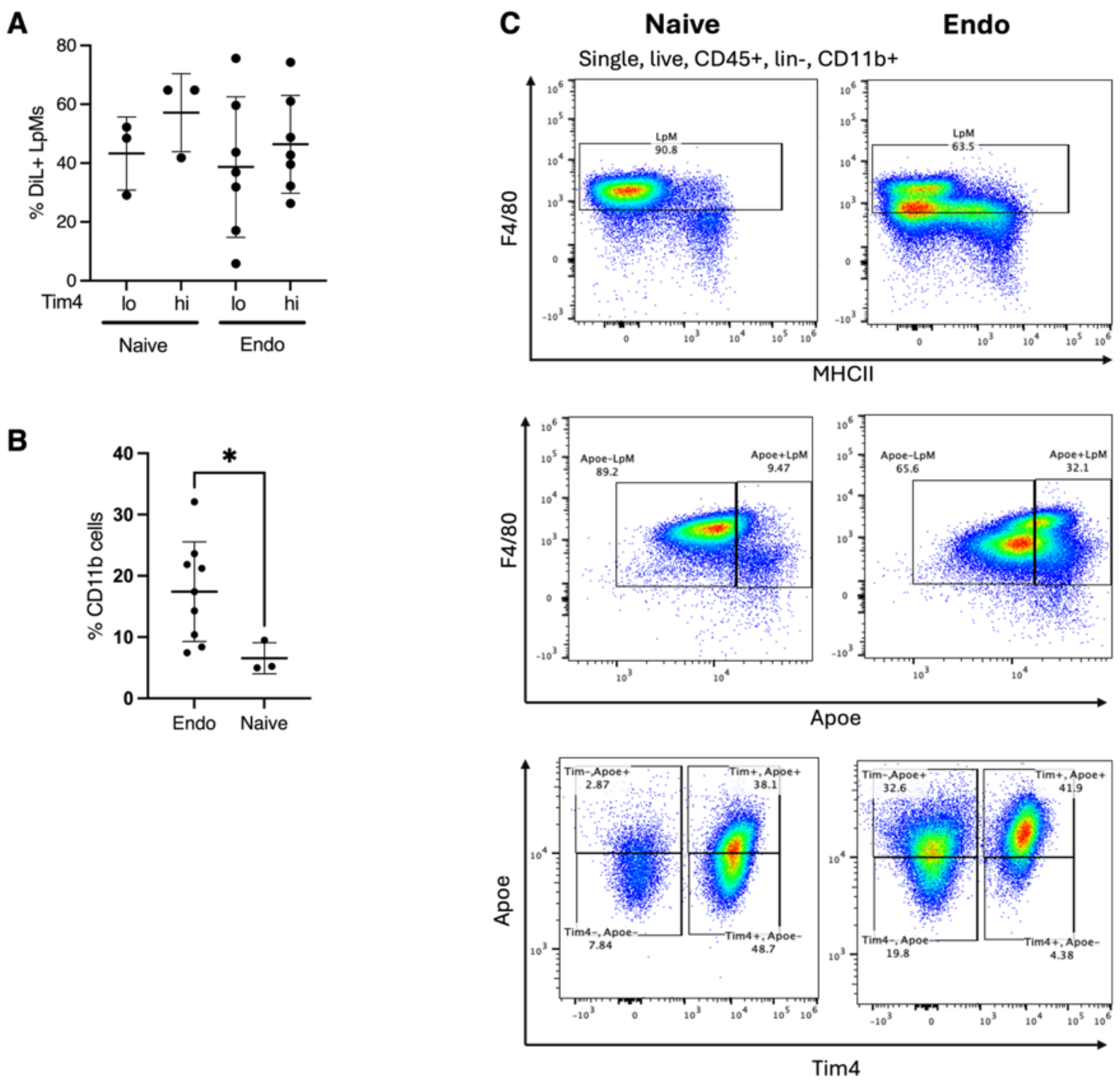

Figure S7: Lipid uptake in LpM and Apoe staining. A) Quantification of lipid uptake by LpM. B) Quantification of Apoe+ LpM. C) Flow plots of showing Apoe staining in LpM derived from mice with (n=9) and without endometriosis (n=3). Statistical tests were performed using a Mann Whitney tests or Kruskal-Wallis. *:p<0.05.

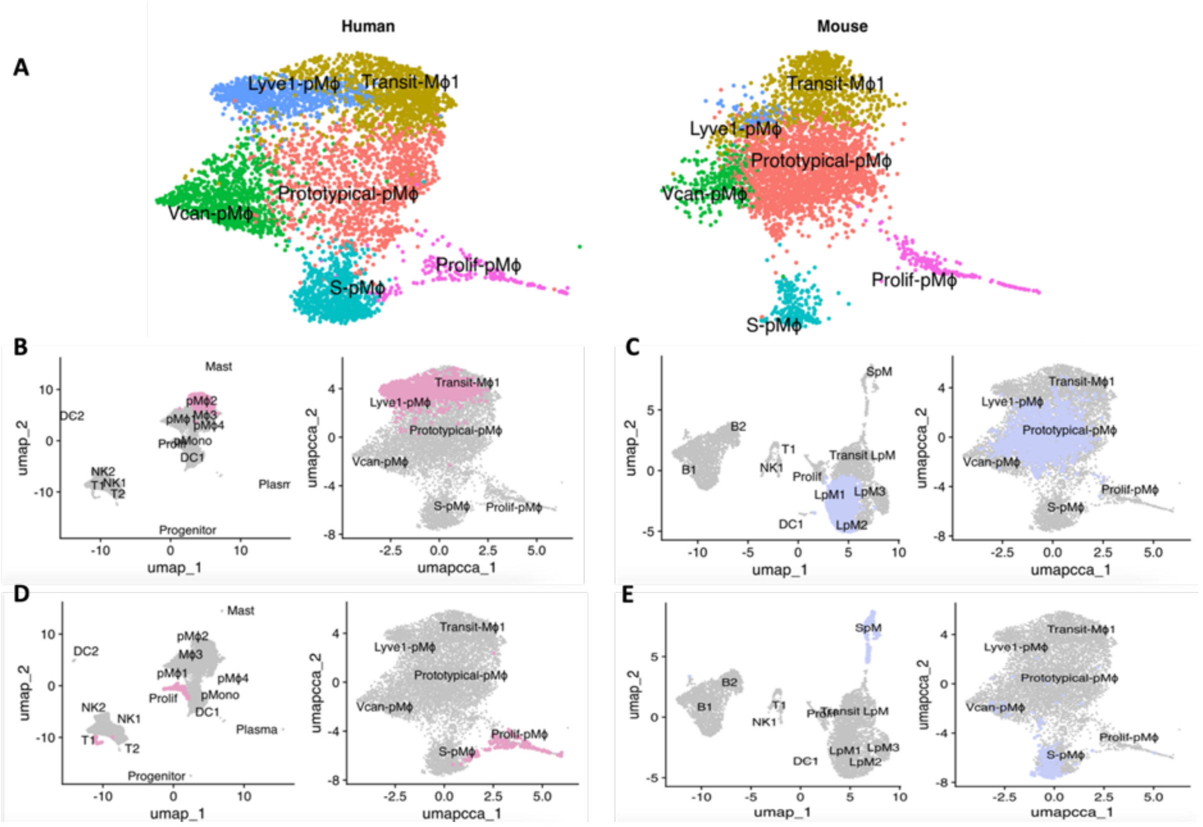

Figure S8: Validation of integrative clustering. A) Integrated UMAP separated by species. B) Human pM2 cells mapping to Lyve+ pM and Transit pM clusters in the integrated map. C) Mouse LpM1 (prototypical) cells mapping to the prototypical pM cluster in the integrated map. D) Human proliferating pM cells mapping to the corresponding prolif pM cluster in the integrated UMAP. E) Mouse SpM cells mapping to integrated (S) pM integrated cluster.

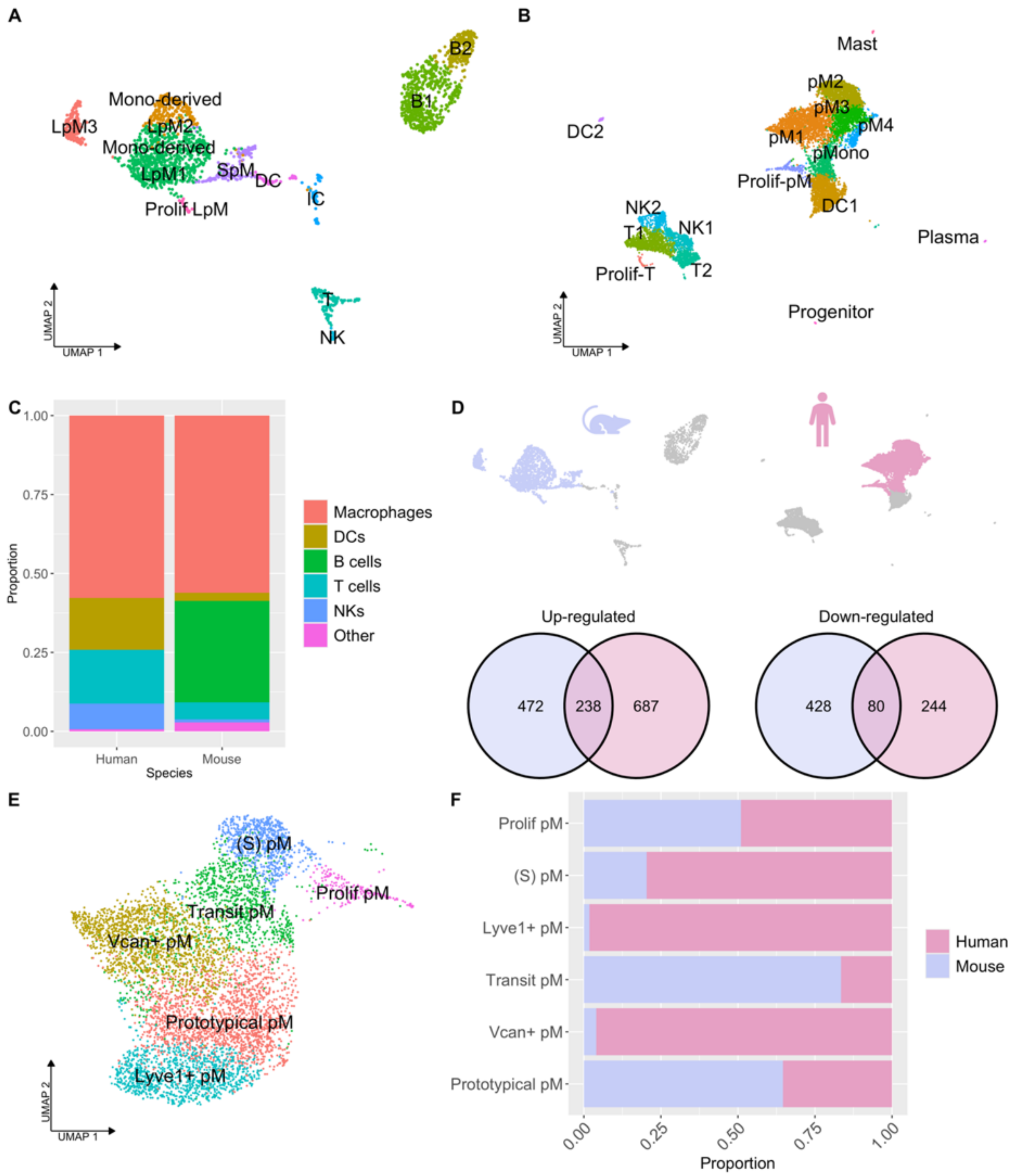

Figure.S9. Cross-species mapping of mouse (intact model) and human peritoneal macrophages. A) UMAP projection of CD45+ cell derived from mouse peritoneal fluid (Endo-Intact), B) UMAP projection of CD45+ cells derived from human peritoneal fluid (Zou et al publicly available dataset). C) Bar chart showing the proportions of each cell type present in the two datasets. The 'other' population includes other cells excluding macrophages, DC, T and NK (e.g B cells, mast cells). D) The macrophage subset was extracted from each dataset (lilac and pink for mouse and human, respectively) and evaluated for shared up- and down-regulated genes, see Venn diagrams. Significance levels for shared up and down-regulated genes were ($p<0.0001$) E) Cross species integration of single-cell RNA-sequencing data was

performed to map mouse and human macrophage subpopulations. F) Bar chart showing cluster member proportions for each species.

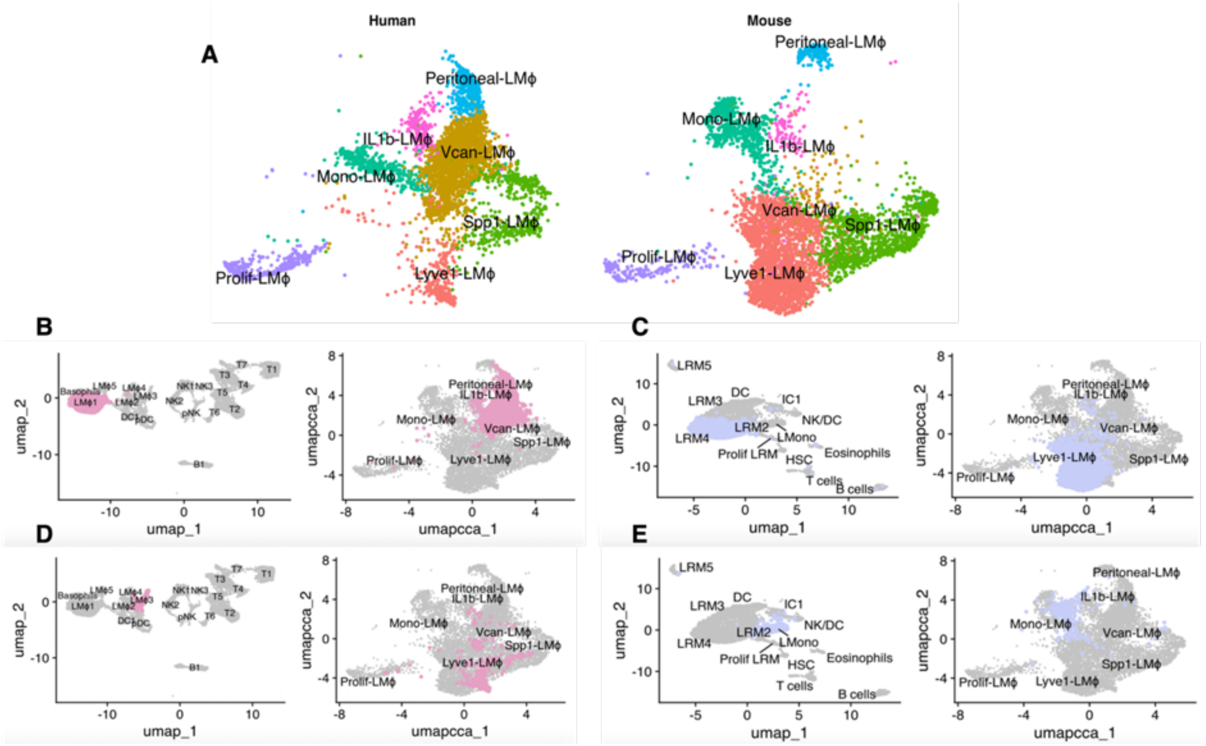

Figure S10: Validation of integrative clustering. A) Integrated UMAP separated by species. B) Human LM1 cells mapping to the Peritoneal LM and Vcan+ LM clusters in the integrated map. C) Mouse LRM4 cells mapping to the Lyve1 cluster in the integrated map. D) Human LM3 cells mapping diffusely onto Vcan+ LM, Lyve+ LM and Spp1+ LMin the integrated UMAP. E) Mouse LMono cells mapping to Lmono integrated cluster.

SI Table 1: Antibodies and applications

Antibody	Fluoro-chrome	Dilution	Cell Type	Supplier + Product Code	Clone	Application
CD3	APC	1:300	T cells	Biolegend 100235	17A2	GFP+/GFP- sorting from MacGreen recipient mice
CD19	APC	1:300	B cells	Biolegend 152410	1D3/CD19	
CD335	APC	1:300	NK cells	Biolegend 137607	29A1.4	
Ly6G	APC	1:300	Neutrophils	Biolegend 127613	1A8	
Siglec F	APC	1:300	Eosinophils	Biolegend 155507	S17007L	
CD45	PerCP Cy5.5	1:200	Leukocytes	Biolegend 103132	30-F11	
F4/80	PE-Cy7	1:200	LpMs	Biolegend 123113	BM8	
CD3	AF700	1:300	T cells	Biolegend 100215	17A2	Analysis of peritoneal fluid macrophages from Apoe mimetic treated mice
CD19	AF700	1:300	B cells	Biolegend 152413	1D3/CD19	
Nk1.1	AF700	1:300	NK cells	Biolegend 156511	S17016D	
Ly6G	AF700	1:300	Neutrophils	Biolegend 127621	1A8	
Siglec F	AF700	1:300	Eosinophils	Biolegend 155533	S17007L	
CD45	Pacific Blue	1:100	Leukocytes	Biolegend 103125	30-F11	
F4/80	PE-Cy7	1:100	LpMs	Biolegend 123113	BM8	
MHC II	PE-Cy5	1:100	SpMs	Biolegend 107611	M5/114.15.2	
Timd4	PE	1:200	Tissue resident LpMs	Biolegend 130005	RMT4-54	
Ly6C	APC-Cy7	1:100	Monocytes	Biolegend 128025	HK1.4	
MHC II	APC-Cy7	1:200	SpMs	Biolegend 107627	M5/114.15.2	Sorting of SAM and TAM-like macrophages
CD3	AF700	1:300	T cells	Biolegend 100215	17A2	
CD19	AF700	1:300	B cells	Biolegend 152413	1D3/CD19	
Nk1.1	AF700	1:300	NK cells	Biolegend 156511	S17016D	
Ly6G	AF700	1:300	Neutrophils	Biolegend 127621	1A8	
Siglec F	AF700	1:300	Eosinophils	Thermo Fisher 56-1702-80	1RNM44N	

Siglec F	PE/Dazzle	1:200	Eosinophils	Biolegend 155529	S17007L	
FOLR2	APC	1:200	TAMs	Biolegend 153305	10/FR2	
F4/80	PE-Cy7	1:100	LpMs	Biolegend 123113	BM8	
CD206	PE	1:100	TAMs	BD Biosciences 568273	Y17-505	
TREM2	FITC	1:30	SAMs	Abcam 119852	YB2/0	
CD11b	e-Fluor 506	1:200	Macrophage /Monocyte	eBioscience 69-0112-80	M1/70	
CD9	Vio-blue	1:50	SAMs	Miltenyi 130-102-745	MZ3	
F4/80	PE-Cy7	1:200	LpMs	Biolegend 123113	BM8	Sorting macrophages for phagocytosis assays
MHC II	PE-Cy5	1:200	SpMs	Biolegend 107611	M5/114.15.2	
CD45	PerCP-Cy5.5	1:100	Leukocytes	Biolegend 103132	30-F11	
Timd4	PE-Cy7	1:200	Tissue resident LpMs	Biolegend 130009	RMT4-54	
CD3	FITC	1:300	T cells	Biolegend 100203	17A2	
CD19	FITC	1:300	B cells	Biolegend 152403	1D3/CD19	
CD335	FITC	1:300	NK Cells	Biolegend 137605	29A1.4	
Ly6G	FITC	1:300	Neutrophils	Biolegend 127605	1A8	
Siglec F	FITC	1:300	Eosinophils	Biolegend 155503	S17007L	
CD45	Pacific Blue	1:100	Leukocytes	Biolegend 103125	30-F11	Sorting CD45+ cells for single cell sequencing
Zombie UV	-	1:100	Live/dead marker	Biolegend 423107	-	Various applications
Fixable Viability stain 660	-	1:100	Live/dead marker	BD Biosciences 564405	-	Various applications
Fixable Viability stain 780	-	1:100	Live/dead marker	BD Biosciences 565388	-	Various applications
CD11b	PECF-594	1:300	Macrophage /Monocyte	Biolegend 101256	M1/70	Various applications
F4/80	APC Fire 750	1:300	Macrophage	Biolegend 123152	-	Apoe Intracellular staining
MHCII	BV605	1:300	SpMs	Biolegend 107639	-	

Tim4	AF647	1:300	Macrophages	Biolegend 130007	-	
Apoe	PE	5:300	Apoe	Biolegend 803404	-	

SI Table 2: Cell ranger metrics

Sample	Estimated Number of Cells	Mean Reads per Cell	Median Genes per Cell	Sequencing Saturation
**Endo-OVX Dataset**				
Menses-like endometrium	1306	393957	2050	93.7%
Sham PF	5645	97631	1326	90.6%
Endo-Ovx PF	6720	77378	1702	84.4%
Lesions	6006	81492	1160	92.0%
**Endo-Intact Dataset**				
Naïve PF	4612	148623	1304	92.8%
Endo-Intact PF	3069	235681	1133	94.8%

References

1. Sasmono, R.T. *et al.* A macrophage colony-stimulating factor receptor-green fluorescent protein transgene is expressed throughout the mononuclear phagocyte system of the mouse. *Blood* **101**, 1155-1163 (2003).

2. Dorning, A. *et al.* Bioluminescent imaging in induced mouse models of endometriosis reveals differences in four model variations. *Dis Model Mech* **14** (2021).

3. Cousins, F.L. *et al.* Evidence from a Mouse Model That Epithelial Cell Migration and Mesenchymal-Epithelial Transition Contribute to Rapid Restoration of Uterine Tissue Integrity during Menstruation. *PLoS One* **9**, e86378 (2014).

4. Greaves, E. *et al.* A novel mouse model of endometriosis mimics human phenotype and reveals insights into the inflammatory contribution of shed endometrium. *Am J Pathol* **184**, 1930-1939 (2014).

5. Hogg, C. *et al.* Macrophages inhibit and enhance endometriosis depending on their origin. *Proc Natl Acad Sci U S A* **118** (2021).

6. Zappia, L. & Oshlack, A. Clustering trees: a visualization for evaluating clusterings at multiple resolutions. *GigaScience* **7** (2018).

7. Tan, Y. *et al.* Single cell analysis of endometriosis reveals a coordinated transcriptional program driving immunotolerance and angiogenesis across eutopic and ectopic tissues. *bioRxiv*, 2021.2007.2028.453839 (2021).

8. Zou, G. *et al.* Cell subtypes and immune dysfunction in peritoneal fluid of endometriosis revealed by single-cell RNA-sequencing. *Cell Biosci* **11**, 98 (2021).

9. Heumos, L. *et al.* Best practices for single-cell analysis across modalities. *Nature Reviews Genetics* **24**, 550-572 (2023).

10. Germain, P.L., Lun, A., Garcia Meixide, C., Macnair, W. & Robinson, M.D. Doublet identification in single-cell sequencing data using scDblFinder. *F1000Res* **10**, 979 (2021).

11. Young, M.D. & Behjati, S. SoupX removes ambient RNA contamination from droplet-based single-cell RNA sequencing data. *Gigascience* **9** (2020).

12. Song, Y., Miao, Z., Brazma, A. & Papatheodorou, I. Benchmarking strategies for cross-species integration of single-cell RNA sequencing data. *Nat Commun* **14**, 6495 (2023).

13. Browaeys, R., Saelens, W. & Saeys, Y. NicheNet: modeling intercellular communication by linking ligands to target genes. *Nature Methods* **17**, 159-162 (2020).

14. Fassbender, A. *et al.* World Endometriosis Research Foundation Endometriosis Phenome and Biobanking Harmonisation Project: IV. Tissue collection, processing, and storage in endometriosis research. *Fertil Steril* **102**, 1244-1253 (2014).

15. Vitonis, A.F. *et al.* World Endometriosis Research Foundation Endometriosis Phenome and Biobanking Harmonization Project: II. Clinical and covariate phenotype data collection in endometriosis research. *Fertil Steril* **102**, 1223-1232 (2014).

16. Greaves, E. *et al.* Estrogen receptor (ER) agonists differentially regulate neuroangiogenesis in peritoneal endometriosis via the repellent factor SLIT3. *Endocrinology*, en20141086 (2014).

17. Greaves, E. *et al.* Estradiol Is a Critical Mediator of Macrophage-Nerve Cross Talk in Peritoneal Endometriosis. *Am J Pathol* (2015).
